# A novel ruthenium complex with 5-fluorouracil suppresses colorectal cancer stem cells by inhibiting Akt/mTOR signaling

**DOI:** 10.1038/s41420-023-01759-6

**Published:** 2023-12-16

**Authors:** Valdenizia R. Silva, Luciano de S. Santos, Maria V. L. de Castro, Rosane B. Dias, Ludmila de F. Valverde, Clarissa A. G. Rocha, Milena B. P. Soares, Claudio A. Quadros, Rodrigo S. Correa, Alzir A. Batista, Daniel P. Bezerra

**Affiliations:** 1 Gonçalo Moniz Institute, Oswaldo Cruz Foundation (IGM-FIOCRUZ/BA), Salvador, Bahia 40296-710 Brazil; 2 Department of Propedeutics, School of Dentistry of the Federal University of Bahia, Salvador, Bahia 40110-909 Brazil; 3 SENAI Institute of Innovation (ISI) in Health Advanced Systems, University Center SENAI/CIMATEC, Salvador, Bahia 41650-010 Brazil; 4 São Rafael Hospital, Rede D’Or/São Luiz, Salvador, Bahia 41253-190 Brazil; 5 Bahia State University, Salvador, Bahia 41150-000 Brazil; 6 Department of Chemistry, Federal University of Ouro Preto, Ouro Preto, Minas Gerais 35400-000 Brazil; 7 Department of Chemistry, Federal University of São Carlos, São Carlos, São Paulo 13561-901 Brazil

**Keywords:** Cancer stem cells, Drug development

## Abstract

[Ru(5-FU)(PPh_3_)_2_(bipy)]PF_6_ (Ru/5-FU) is a novel ruthenium complex with 5-fluorouracil with promising potential against colorectal cancer (CRC). In the present study, we investigated the molecular mechanism of Ru/5-FU action in HCT116 CRC cells. Ru/5-FU exhibited potent cytotoxicity on a panel of cancer cell lines and on primary cancer cells and induced apoptosis in HCT116 CRC cells. Ru/5-FU reduced *AKT1* gene transcripts, as well as the expression of Akt1 and Akt (pS473) and downstream Akt proteins mTOR (pS2448), S6 (pS235/pS236), 4EBP1 (pT36/pT45), GSK-3β (pS9) and NF-κB p65 (pS529), but not Akt upstream proteins Hsp90 and PI3K p85/p55 (pT458/pT199), indicating an inhibitory action of Akt/mTOR signaling. Ru/5-FU increased LC3B expression and reduced p62/SQSTM1 levels, indicating autophagy induction. Curiously, the autophagy inhibitors 3-methyladenine and chloroquine increased Ru/5-FU-induced cell death, indicating an induction of cytoprotective autophagy by this compound. Ru/5-FU also reduced clonogenic survival, as well as the percentage of CD133+ cells and colonosphere formation, indicating that Ru/5-FU can suppress stem cells in HCT116 cells. Ru/5-FU inhibited cell migration and invasion in wound healing assays and Transwell cell invasion assays, along with a reduction in vimentin expression and an increase in E-cadherin levels, indicating that Ru/5-FU can interfere with epithelial-mesenchymal transition. Ru/5-FU also inhibited in vivo HCT116 cell development and experimental lung metastases in mouse xenograft models. Altogether, these results indicate that Ru/5-FU is an anti-CRC chemotherapy drug candidate with the ability to suppress stemness in CRC cells by inhibiting Akt/mTOR signaling.

## Introduction

Colorectal cancer (CRC) represents a serious public health problem, being the third most diagnosed type of cancer and the second cause of death from cancer. In 2020, 1.9 million new cases and 935 thousand deaths were related to CRC worldwide [1]. The chemotherapeutic 5-fluorouracil (5-FU), introduced in the clinical treatment of CRC in 1957, is still the recommended first-line treatment for advanced CRC and has achieved response rates of 10%‐15%, which can be improved to approximately 40%‐50% when combined with irinotecan and oxaliplatin [2, 3]. Some targeted therapies are used in the second line of treatment for metastatic CRC, and despite some advances in CRC therapies, mortality has remained relatively stable in recent decades, indicating that new treatments are urgently needed.

According to the modern concept of cancer biology, cancer is a heterogeneous disease formed by a small subpopulation of cells called cancer stem cells (CSCs) that have pluripotency and self-renewal capacity. These cells are directly associated with disease progression, recurrence and drug resistance [4–6]. In particular, current chemotherapies decrease the tumor mass and enrich CSCs in residual tumors, contributing to recurrence [7–9]. Therefore, CRC therapy must eradicate cells with CSC properties for a good clinical outcome [4].

**Fig. 1:**
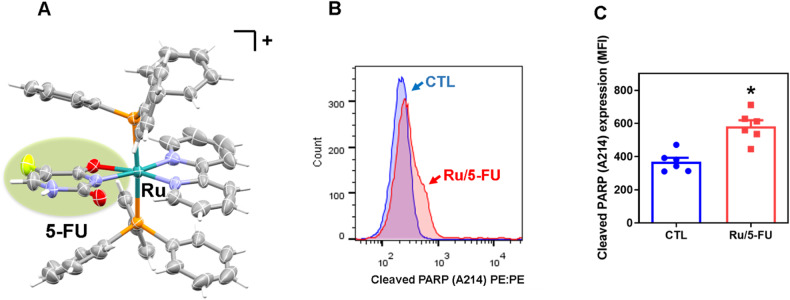
Ru/5-FU induces apoptotic cell death. **A** Chemical structure of Ru/5-FU. **B**, **C** Quantification of PARP-1 expression in HCT116 cells after 24 h of incubation with 4 μM Ru/5-FU, as determined by flow cytometric analysis. The vehicle (0.2% DMSO) was used as a control (CTL). Data are shown as the mean ± S.E.M. of three independent experiments carried out in duplicate. * *P* < 0.05 compared to CTL by Student’s *t* test. MFI: Mean fluorescence intensity.

Recently, our research group synthesized a novel molecule combining a ruthenium-based complex with chemotherapeutic 5-FU (Ru/5-FU) with the molecular formula [Ru(5-FU)(PP_h3_)_2_(bipy)]PF_6_ (Fig. 1A). This novel ruthenium-based 5-FU complex was more potent than 5-FU in 2D and 3D cell cultures and caused caspase-mediated apoptosis in human CRC HCT116 cells [10]. In the present study, we investigated the molecular mechanism of action of Ru/5-FU in HCT116 CRC cells. We found that Ru/5-FU can suppress CRC stemness by inhibiting Akt/mTOR signaling.

## Results

### Ru/5-FU exhibits potent cytotoxicity in a panel of cancer cells and induces apoptosis in HCT116 CRC cells

Ru/5-FU exhibited potent cytotoxicity on a panel of 24 cancer cell lines (HCT116, HepG2, NB4, THP-1, JUKART, K-562, HL-60, KG-1a, MDA-MB-231, MCF-7, 4T1, HSC-3, CAL 27, SCC-25, SCC4, SCC-9, A549, H1299, PANC-1, OVCAR-3, DU 145, U-87 MG, A-375, and B16-F10), demonstrating IC_50_ values ranging from 1.2 to 9.2 µM for OVCAR and MCF-7 cells, respectively, while showing IC_50_ values of 7.1, 8.9 and 3.1 µM for noncancerous MRC-5, PBMC and BJ cells, respectively (Table S1). Selectivity indexes were calculated and are shown in Table S2.

Doxorubicin (DOX) had IC_50_ values ranging from 0.03 in JUKART to 18.6 µM for KG-1a and had IC_50_ values of 2.8, 1.3 and 0.7 µM for noncancerous MRC-5, PBMC and BJ cells, respectively. 5-FU had IC_50_ values ranging from 5.2 in THP-1 cells and values greater than 192 µM for MCF-7 and HSC-3 cells and had IC_50_ values of 31.3 for noncancerous MRC-5 cells and values greater than 192 µM for PBMCs and BJ cells.

To determine whether Ru/5-FU also causes cytotoxicity in primary cancer cells, we cultured two histologic types of CRC, cholangiocarcinoma and papilliferous malignant mesothelioma cells, and treated them with 25 μg/mL Ru/5-FU (27.4 μM) (Fig. S1). Cell viability was reduced by 70.1, 69.1, 81.2 and 78.4%, respectively, after Ru/5-FU treatment, while DOX, at 25 μg/mL (46 μM), reduced cell viability by 71.0, 36.2, 65.6 and 64.3%, respectively. 5-FU at 25 μg/mL (192.2 μM) reduced cell viability by 4.1, 6.8, 54.8 and 61.7%, respectively.

**Fig. 2:**
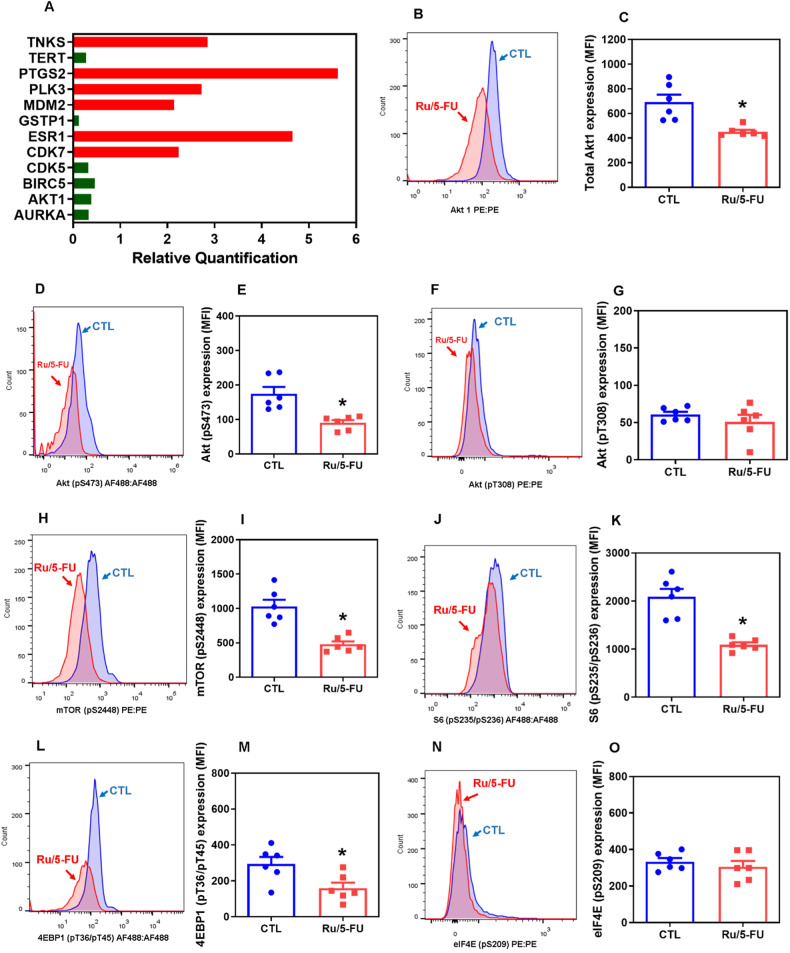
Ru/5-FU suppresses Akt/mTOR signaling. **A** Genes up- and downregulated in HCT116 cells after 12 h of treatment with 4 µM Ru/5-FU. The vehicle (0.2% DMSO) was used as a control (CTL). Data are shown as relative quantification (RQ) compared to CTL. The genes were considered to be upregulated if RQ ≥ 2 (red bars) and downregulated if RQ ≤ 0.5 (green bars). Quantification of Akt1 (**B**, **C**), Akt (pS473) (**D**, **E**), Akt (pT308) (**F**, **G**), mTOR (pS2448) (**H**, **I**), S6 (pS235/pS236) (**J**, **K**), 4EBP1 (pT36/pT45) (**L**, **M**), and elF4E (pS209) (**N**, **O**) expression in HCT116 cells after 24 h of incubation with 4 μM Ru/5-FU, as determined by flow cytometric analysis. The vehicle (0.2% DMSO) was used as a control (CTL). Data are shown as the mean ± S.E.M. of three independent experiments carried out in duplicate. *P < 0.05 compared to CTL by Student’s *t* test. MFI: Mean fluorescence intensity.

Since Ru/5-FU was able to reduce the viability of primary CRC cells, we decided to investigate the molecular mechanisms of action of Ru/5-FU in HCT116 CRC cells. Initially, we detected that the levels of PARP cleavage (Asp214) (Fig. 1B, C) were increased and the expression of *BIRC5* and *CDK5* genes (Fig. 2A and Table S3) was reduced in Ru/5-FU-treated HCT116 cells, indicating the presence of apoptotic cell death. The role of the proapoptotic protein BAD in Ru/5-FU-induced cell death was also evaluated using the BAD KO SV40 MEF cell line compared to its parental cell line WT SV40 MEF (Figs. S2–S5). On the other hand, Ru/5-FU caused BAD-independent cell death.

### Ru/5-FU inhibits Akt/mTOR signaling in HCT116 CRC cells

By analyzing the transcripts of 82 target genes using a qPCR array, we identified that Ru/5-FU can downregulate *AKT1* (RQ = 0.392) in CRC HCT116 cells, suggesting that disruption of protein kinase B (Akt) signaling is a molecular target for Ru/5-FU (Fig. 2A and Table S3). To confirm this hypothesis, we measured the expression of the Akt protein and its upstream and downstream proteins. The expression of Akt1 and Akt (pS473) was decreased in Ru/5-FU-treated cells (Fig. 2B–G). The expression levels of the downstream Akt proteins mTOR (pS2448), S6 (pS235/pS236), and 4EBP1 (pT36/pT45) also decreased after Ru/5-FU treatment (Fig. 2H–O). On the other hand, the levels of the Akt upstream proteins Hsp90 and PI3K p85/p55 (pT458/pT199) did not change in Ru/5-FU-treated cells (Figure S6). These findings indicate that Ru/5-FU can suppress Akt/mTOR signaling in HCT116 CRC cells.

Another downstream Akt protein is glycogen synthase kinase 3 (GSK-3). Akt inhibits GSK-3 activity via phosphorylation of Ser-21 in GSK-3α or Ser-9 in GSK-3β. GSK-3β is an antagonist of Wnt signaling. We found that Ru/5-FU reduced GSK-3β (pS9) expression in HCT116 CRC cells (Fig. S7). Therefore, we hypothesized that Ru/5-FU can inhibit Wnt signaling. However, cotreatment with lithium chloride, a Wnt activator, was not able to prevent Ru/5-FU-induced cell death in HCT116 CRC cells (Fig. S8).

**Fig. 3:**
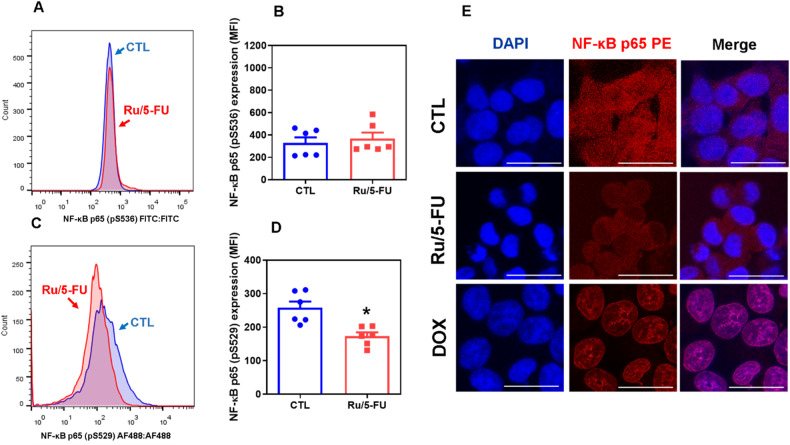
Ru/5-FU inhibits NF-κB signaling. Quantification of NF-κB p65 (pS536) (**A**, **B**) and NF-κB p65 (pS529) (**C**, **D**) expression in HCT116 cells after 24 h of incubation with 4 μM Ru/5-FU, as determined by flow cytometric analysis. The vehicle (0.2% DMSO) was used as a control (CTL). Data are shown as the mean ± S.E.M. of three independent experiments carried out in duplicate. *P < 0.05 compared to CTL by Student’s *t* test. MFI: Mean fluorescence intensity. **E** Representative immunofluorescence images of NF-κB p65 in HCT116 cells after 24 h of incubation with 4 μM Ru/5-FU. Scale bar = 25 μm.

Akt also stimulates Ikk, which inhibits IκB and triggers the NF-κB signaling pathway. Therefore, we assessed whether Ru/5-FU can affect the NF-κB pathway. Although Ru/5-FU did not change NF-κB p65 (pS536) expression (Fig. 3A, B), a reduction in NF-κB p65 (pS529) (Fig. 3C, D) expression was observed in Ru/5-FU-treated HCT116 CRC cells. Moreover, a reduction in nuclear NF-κB p65 protein was observed in Ru/5-FU-treated CRC HCT116 cells, indicating that this compound interferes with NF-κB p65 signaling (Fig. 3E). DOX, an NF-κB p65 activator used as a control, increased nuclear NF-κB p65 protein in HCT116 CRC cells.

Akt can also increase nuclear Mdm2 stability, decreasing p53 levels. Thus, we hypothesized that Ru/5-FU can activate p53 signaling. The expression of H2AX (pS139), Mdm2 and p53 (pS15) was quantified in Ru/5-FU-treated HCT116 CRC cells. However, although Ru/5-FU increased the expression level of the DNA damage marker H2AX (pS139), no significant changes in Mdm2 and p53 (pS15) levels were detected (Fig. S9). Moreover, pharmacologic inhibition of p53 with pifithrin-α did not prevent cell death induced by Ru/5-FU in HCT116 CRC cells, indicating p53-independent cell death (Fig. S10).

Akt is linked to mitogen-activated protein kinase (MAPK) signaling through epidermal growth factor receptor (EGFR). Therefore, we decided to check whether Ru/5-FU can affect MAPK signaling. The expression levels of the three main MAPK proteins, JNK2 (pT183/pY185), p38α (pT180/pY182), and ERK1 (pT202/pY204), were determined in Ru/5-FU-treated HCT116 CRC cells after acute (15 and 30 min) and prolonged (24 h) incubation. The level of JNK2 (pT183/pY185) increased after 15 min of incubation with Ru/5-FU (Fig. S11). However, coincubation with a JNK inhibitor (SP 600125), a p38 MAPK inhibitor (PD 169316), or an inhibitor (U-0126) of mitogen-activated protein kinase kinase (MKK, which inhibits the activation of ERK1/2) did not prevent Ru/5-FU-induced cell death in CRC HCT116 cells (Fig. S12), indicating that MAPK signaling is not essential for Ru/5-FU-induced cell death.

### Ru/5-FU induces autophagy in HCT116 CRC cells

**Fig. 4:**
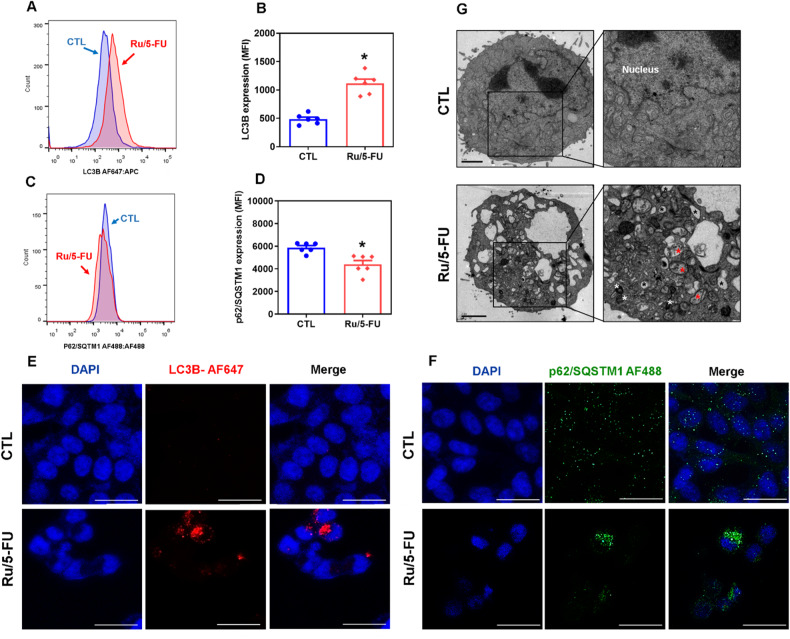
Ru/5-FU induces autophagic process. Quantification of LC3B (**A**, **B**) and p62/SQSTM1 (**C**, **D**) expression in HCT116 cells after 24 h of incubation with 4 μM Ru/5-FU, as determined by flow cytometric analysis. The vehicle (0.2% DMSO) was used as a control (CTL). Data are shown as the mean ± S.E.M. of three independent experiments carried out in duplicate. *P < 0.05 compared to CTL by Student’s *t* test. MFI: Mean fluorescence intensity. **E** Representative immunofluorescence images of LC3B in HCT116 cells after 24 h of incubation with 4 μM Ru/5-FU. Scale bar = 25 μm. **F** Representative immunofluorescence images of p62/SQSTM1 in HCT116 cells after 24 h of incubation with 4 μM Ru/5-FU. Scale bar = 25 μm. **G** Representative MET images of HCT116 cells after 12 h of incubation with 4 μM Ru/5-FU. Black asterisks represent empty vacuoles, white asterisks represent electron-dense mitochondria, and red asterisks represent autophagic vacuoles. Scale bar = 2 μm.

mTOR is a negative modulator of autophagy. Since Ru/5-FU can reduce mTOR (pS2448) expression in HCT116 CRC cells, we hypothesized that it could induce autophagy. Therefore, we quantified the protein expression levels of LC3B and p62/SQSTM1 in Ru/5-FU-treated HCT116 CRC cells. Ru/5-FU treatment increased LC3B (Fig. 4A, B, E) and reduced p62/SQSTM1 (Fig. 4C, D, F), indicating the induction of autophagy. Furthermore, transmission electron microscopy (TEM) analysis indicated the presence of autophagic vacuoles in Ru/5-FU-treated HCT116 cells (Fig. 4G).

Since autophagy induction can present pleiotropic effects in cancer therapy, we used two autophagy inhibitors to check the effect of Ru/5-FU-induced autophagy on Ru/5-FU-induced cell death in HCT116 CRC cells. 3-Methyladenine (3-MA) (Fig. S13), an early-stage autophagy inhibitor that blocks autophagosome formation by inhibiting PI3K, and chloroquine (CQ) (Fig. S14), a lysosomotropic agent that prevents fusion between autophagosomes and lysosomes in the final stage of autophagy, were used to block autophagy in Ru/5-FU-treated cells. Curiously, 3-MA prevented the increase in Ru/5-FU-induced LC3B expression. Both autophagy inhibitors increased the cell death induced by this compound (Figs. S15, S16), indicating that Ru/5-FU induces cytoprotective autophagy in HCT116 CRC cells.

### Ru/5-FU suppresses stemness in HCT116 CRC cells

**Fig. 5:**
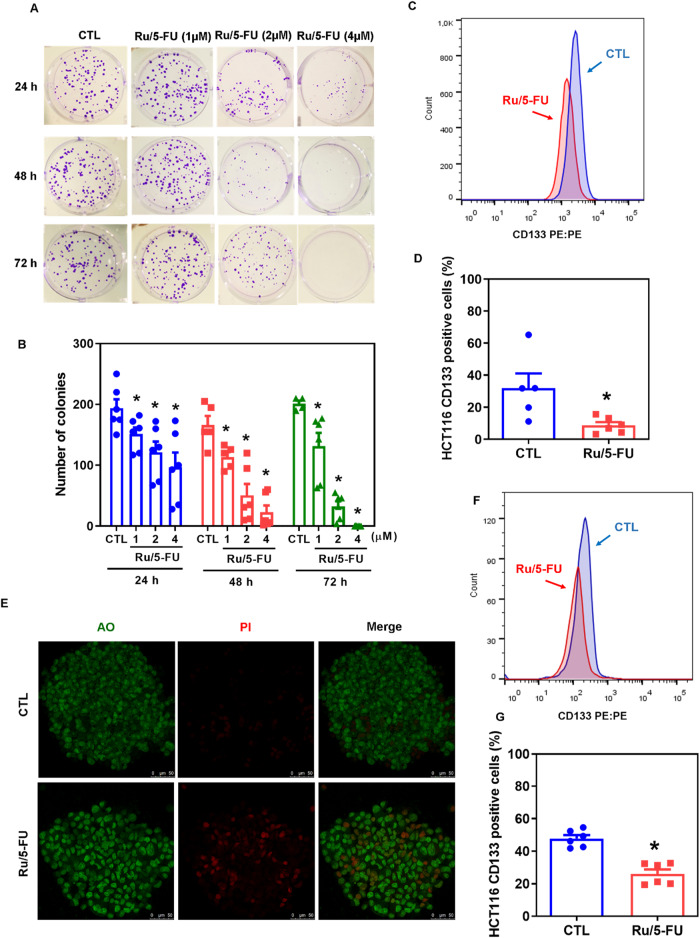
Ru/5-FU inhibits CRC stem cells. **A** Representative images and (**B**) quantification of the number of colonies formed from HCT116 cells after treatment with Ru/5-FU. **C**, **D** Quantification of CD133 expression in HCT116 cells cultured in a monolayer after 24 h of incubation with 4 μM Ru/5-FU, as determined by flow cytometric analysis. **E** Representative confocal images of colonospheres formed from HCT116 cells after 24 h of incubation with 4 μM Ru/5-FU. The cells were stained with acridine orange (AO, green cells) and propidium iodide (PI, red cells that represent dead cells). Scale bar = 50 μm. **F**, **G** Quantification of CD133 expression in HCT116 cells cultured in colonospheres after 24 h of incubation with 4 μM Ru/5-FU, as determined by flow cytometric analysis. The vehicle (0.2% DMSO) was used as a control (CTL). Data are shown as the mean ± S.E.M. of three independent experiments carried out in duplicate. *P < 0.05 compared to CTL by Student’s *t* test or one-way ANOVA followed by Dunnett’s multiple comparisons test.

Both the Akt/mTOR and NF-κB signaling pathways have been reported as molecular targets to eliminate CRC stem cells. Therefore, we hypothesized that Ru/5-FU could suppress stemness in HCT116 CRC cells. First, we performed a long-term colony formation assay to determine whether Ru/5-FU can affect the clonogenic ability of HCT116 CRC cells. Interestingly, treatment with Ru/5-FU significantly reduced the clonogenic survival of HCT116 cells in a concentration- and time-dependent manner (Fig. 5A, B). In addition, Ru/5-FU reduced the percentage of HCT116 CD133+ cells, a CRC stem cell subpopulation (Fig. 5C, D).

Next, we tested the effect of Ru/5-FU on colonospheres formed from HCT116 cells. Likewise, Ru/5-FU reduced colonosphere formation in a concentration- and time-dependent manner (Fig. S17), indicating that this compound can suppress stemness in HCT116 CRC cells. Furthermore, an increase in dead cells was detected in Ru/5-FU-treated colonospheres (Fig. 5E). A reduction in HCT116 CD133+ cells in Ru/5-FU-treated colonospheres was also found (Fig. 5F, G).

### Ru/5-FU inhibits epithelial-mesenchymal transition in HCT116 CRC cells

**Fig. 6:**
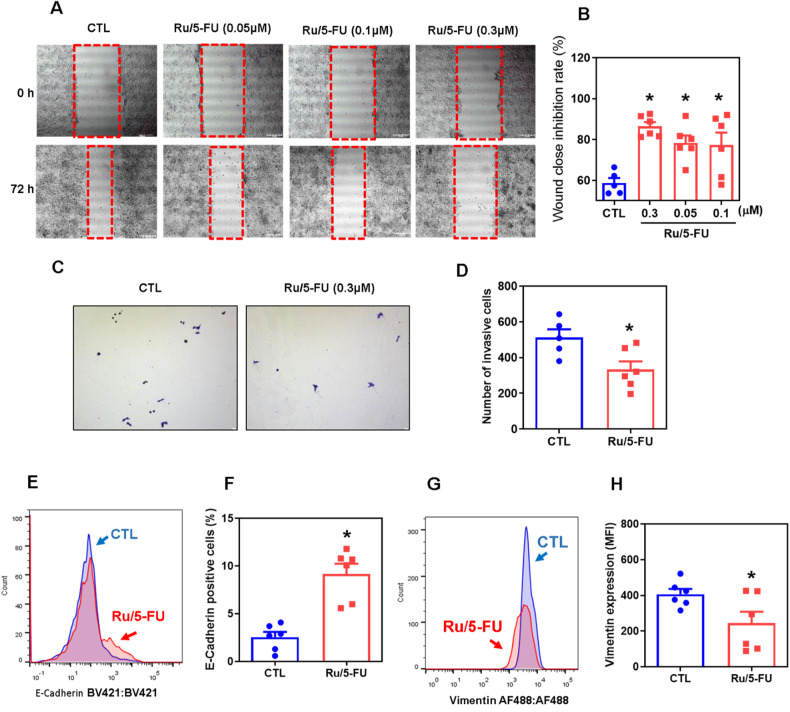
Ru/5-FU suppresses cell motility. **A** Representative images and (**B**) quantification of HCT116 cell migration in the wound healing assay after 72 h of incubation with Ru/5-FU. **C** Representative images and (**D**) quantification of HCT116 cell invasion in the Transwell invasion assay after 48 h of incubation with Ru/5-FU. Quantification of E-cadherin (**E**, **F**) and (**G**, **H**) vimentin expression in HCT116 cells after 24 h of incubation with 4 μM Ru/5-FU, as determined by flow cytometric analysis. The vehicle (0.2% DMSO) was used as a control (CTL). Data are shown as the mean ± S.E.M. of three independent experiments carried out in duplicate. *P < 0.05 compared to CTL by Student’s *t* test or one-way ANOVA followed by Dunnett’s multiple comparisons test. MFI: Mean fluorescence intensity.

The CRC stem cell population shares epithelial-mesenchymal transition (EMT)-like cell features, and both are regulated by different cell signaling pathways, including the Akt/mTOR and NF-κB pathways. Therefore, we hypothesized that Ru/5-FU can inhibit EMT in HCT116 CRC cells. First, we observed that Ru/5-FU inhibited cell migration in the wound healing assay (Fig. 6A, B) at noncytotoxic concentrations (Fig. S18) in HCT116 CRC cells. In addition, invasiveness was assessed by Transwell cell invasion assay using chambers coated with Matrigel to simulate invasion through the extracellular matrix. Likewise, Ru/5-FU also inhibited the invasive capacity of HCT116 CRC cells (Fig. 6C, D).

Next, the protein expression levels of two EMT markers, E-cadherin and vimentin, were quantified in HCT116 CRC cells treated with Ru/5-FU. The expression level of E-cadherin was increased (Fig. 6E, F), while the expression level of vimentin was decreased (Fig. 6G, H) in RU/5-FU-treated cells, indicating that this molecule can inhibit EMT in CRC HCT116 cells.

### Ru/5-FU inhibits in vivo HCT116 CRC cell development and experimental lung metastases in mouse xenograft models

**Fig. 7:**
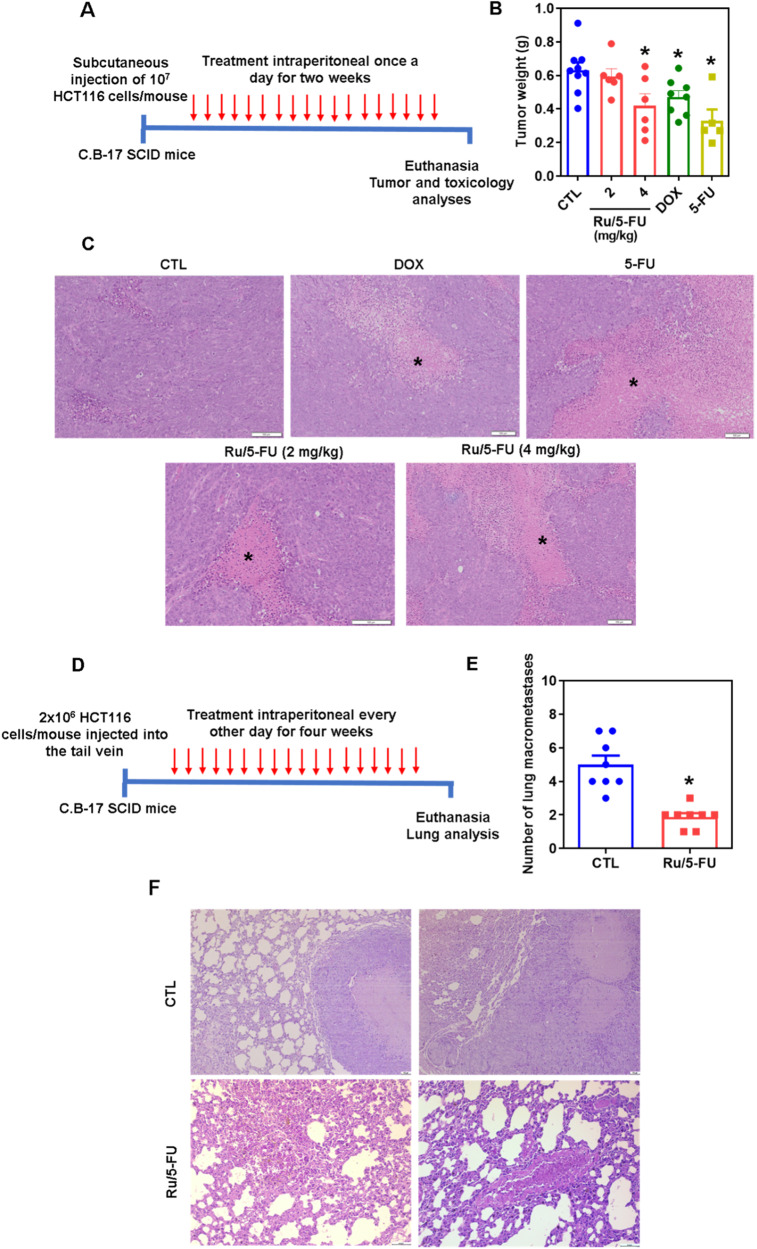
Ru/5-FU exhibits antitumor potential in mouse models. **A** In vivo antitumor experimental design of Ru/5-FU in C. B-17 SCID mice inoculated with HCT116 cells by subcutaneous injection. The animals were treated with Ru/5-FU at doses of 2 and 4 mg/kg intraperitoneally once a day for two weeks. **B** In vivo antitumor activity of Ru/5-FU. The vehicle (5% DMSO) was used as a control (CTL). DOX (0.8 mg/kg) and 5-FU (15 mg/kg) were used as positive controls. Data are presented as the mean ± S.E.M. from 5 to 10 animals. **C** Representative histological analysis of HCT116 tumor tissues stained with hematoxylin and eosin and analyzed by light microscopy. The asterisks indicate areas of tissue necrosis. Scale bar = 100 μm. **D** In vivo antimetastatic experimental design of Ru/5-FU in C. B-17 SCID mice grafted with HCT116 cells by tail vein injection. The animals were treated with 4 mg/kg Ru/5-FU intraperitoneally every other day for four weeks. **E** The in vivo antimetastatic potential of Ru/5-FU. The vehicle (5% DMSO) was used as a control (CTL). Data are presented as the mean ± S.E.M. from 8 animals. **F** Representative histological analysis of lung tissues stained with hematoxylin and eosin and analyzed by light microscopy. Scale bar = 50 or 100 μm. *P < 0.05 compared to CTL by Student’s *t* test or one-way ANOVA followed by Dunnett’s multiple comparisons test.

The in vivo effects of Ru/5-FU were assessed in two different xenograft models: an antitumor model and an experimental lung metastasis model. The in vivo antitumor activity of Ru/5-FU was investigated in C. B-17 SCID mice grafted with HCT116 cells by subcutaneous injection. The animals were treated with Ru/5-FU at doses of 2 and 4 mg/kg intraperitoneally once a day for two weeks (Fig. 7A). At the end of treatment, the mean weight of the tumors in the control group was 630.9 ± 46.8 mg. In animals treated with Ru/5-FU, the mean tumor weights were 595.5 ± 44.5 and 419.0 ± 71.5 mg at lower and higher doses, representing tumor inhibition rates of 5.6 and 33.6%, respectively (Fig. 7B). Fifty percent of animals treated with 5-FU and one animal treated with the highest dose of Ru/5-FU died during treatment. DOX at a dose of 0.8 mg/kg and 5-FU at a dose of 15 mg/kg reduced tumor weight by 25.4% and 47.9%, respectively.

The xenograft tumors displayed highly pleomorphic and proliferative cancer cells distributed in a solid, disorganized growth pattern within a sparse stroma (Fig. 7C). All tumors were classified as poorly differentiated adenocarcinoma. Despite observing areas of coagulative necrosis and inflammatory cells in all treatments, these findings were more extensive in animals treated with the highest dose of Ru/5-FU. Meanwhile, in the control group, the necrotic areas were patchy.

The in vivo antimetastatic potential of Ru/5-FU was investigated in C. B-17 SCID mice grafted with HCT116 cells by tail vein injection (Fig. 7D). The animals were treated with 4 mg/kg Ru/5-FU intraperitoneally every other day for four weeks. The mean number of lung metastases was 1.9 ± 0.2 in Ru/5-FU-treated animals compared to 5.0 ± 0.5 in the control group (Fig. 7E). Histological analysis of the lungs demonstrated the presence of more extensive metastatic nodules (with necrotic centers) and greater numbers in the negative control group than in the Ru/5-FU group (Fig. 7F).

Toxicological parameters were also examined in the animals treated with Ru/5-FU. A decrease in body weight was observed in animals treated with 5-FU and Ru/5-FU at higher doses (*p* < 0.05) (Table S4). No significant changes were observed in organ weights in any group with the exception of the liver of 5-FU-treated animals, which decreased in relation to the control. In the hematological analysis, the number of leukocytes remained unchanged for animals treated with DOX and Ru/5-FU at lower doses; however, a decrease in leukocyte numbers was observed in animals treated with 5-FU and Ru/5-FU at higher doses (Table S5). The number of erythrocytes was not significantly altered in any group.

The histopathological alterations of the organs (liver, kidneys, lungs and heart) were analyzed by optical microscopy (Fig. S19). The hepatic parenchyma was partially preserved by the presence of histological alterations such as moderate hydropic degeneration and focal areas of necrosis due to coagulation of hepatocytes. Moderate vascular hyperemia and focal mixed inflammation were observed in all experimental groups. In addition, the portal architecture ranged from preserved to partially preserved among animals. The renal architecture was preserved in all groups; however, focal areas of coagulation necrosis were observed in the renal cortex tubules of animals treated with the highest dose of Ru/5-FU. In addition, moderate vascular hyperemia and a slight decrease in urinary space due to glomerular hyalinization were observed in all animals. All animals had partially preserved lung architecture due to reduced airspace (areas of atelectasis) and thickening of the alveolar septa. The histopathological changes observed were moderate to severe vascular hyperemia, focal areas of inflammation (polymorphonuclear predominance), mild fibrosis, and focal areas of hemorrhage and hemosiderin accumulation. Furthermore, tumor nodules were observed only in the lungs of the control group. No histological changes were observed in the hearts of any of the experimental groups.

## Discussion

**Fig. 8:**
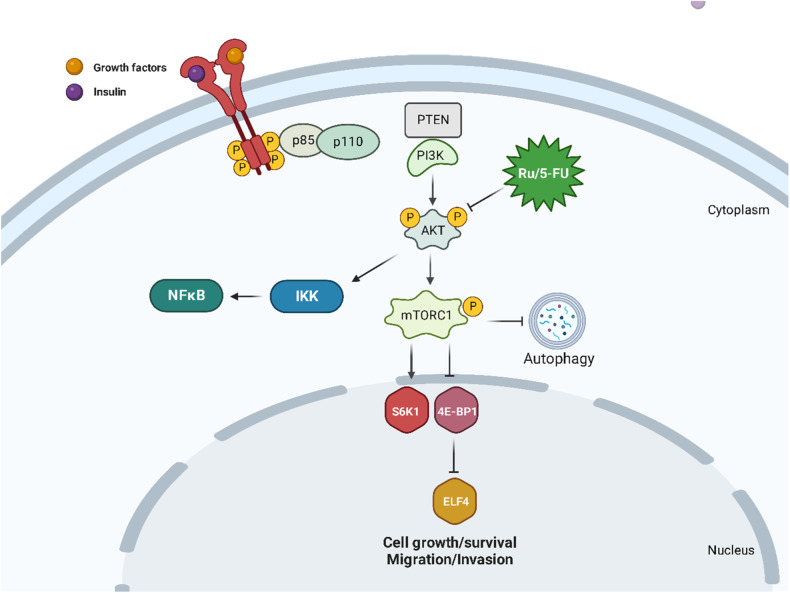
Proposed molecular mechanism of action of RU/5-FU.

In this work, we determined for the first time the molecular mechanism of action of the novel ruthenium-based 5-FU complex Ru/5-FU. It showed potent cytotoxicity against cancer cell lines and primary cancer cells from different histological types. Ru/5-FU suppressed stemness in HCT116 CRC cells via inhibition of Akt/mTOR signaling and triggering apoptosis. Induction of cytoprotective autophagy and inhibition of EMT were also found in Ru/5-FU-treated HCT116 CRC cells. In a mouse xenograft model, Ru/5-FU inhibited CRC HCT116 cell development and experimental lung metastases in vivo. Figure 8 summarizes the molecular mechanism of action of Ru/5-FU.

The induction of apoptotic cell death by Ru/5-FU in HCT116 CRC cells was previously reported [10]. Herein, we detected a significant increase in PARP cleavage (Asp214), an apoptotic feature, as well as a reduction in the gene expression of *BIRC5* and *CDK5*, antiapoptotic molecules, in Ru/5-FU-treated cells, corroborating these results. The ability to induce apoptosis has been reported by different ruthenium complexes against diverse cancer types, including CRC [11–13], leukemia [14, 15], liver cancer [16–18] and breast cancer [19].

Previously, we reported that 5-FU blocks the S phase of the cell cycle in HCT116 cells; however, although Ru/5-FU increased DNA fragmentation, cell accumulation was not detected at any phase of the cell cycle in HCT116 cells treated with Ru/5-FU, indicating a different mechanism of action between Ru/5-FU and 5-FU [10]. Using a q-PCR array, we found that Ru/5-FU can reduce the gene expression level of *AKT1*. Furthermore, we validated these data by quantifying the protein expression levels of some upstream and downstream molecules of the PI3K/Akt/mTOR signaling pathway and concluded that Ru/5-FU inhibits Akt/mTOR signaling and NF-κB signaling. In any case, the gene expression levels of other pathways, including downregulation of *TERT*, which is related to telomerase activity, and *AURKA*, which is related to cell division by regulation of mitosis, were also affected by Ru/5-FU, and they can also contribute to its cytotoxicity. Although the gene expression level of *GSTP1*, an intracellular antioxidant enzyme, was downregulated in Ru/5-FU-treated cells, its cytotoxicity was not associated with oxidative stress, as previously reported [10].

Ruthenium(II)-cyclopentadienyl-derived complexes were reported to induce apoptosis in CRC RKO and SW480 cell lines via inhibition of the MEK/ERK and PI3K/Akt signaling pathways [20]. Likewise, cyclometalated Ru(II) β-carboline complexes led to apoptotic cell death in HeLa cervical cancer cells through the inhibition of ERK and Akt signaling [21]. Cyclometalated Ru(II)-isoquinoline complexes caused apoptosis in cisplatin-resistant lung cancer A549/DDP cells by modulating Akt/GSK-3β/Fyn signaling [22]. A ruthenium(III)-pyrimidine Schiff base complex induced apoptotic cell death in liver cancer HepG2 cells along with downregulation of the transcripts of the mTOR/Akt and NF-κB genes [23].

Ruthenium complexes with piplartine induced apoptosis mediated by MAPKs (JNK/p38/ERK1/2) in HCT116 cells by a p53-dependent pathway [11]. Silva et al. [12] described that a Ru(II)-thymine complex causes DNA damage and apoptotic cell death through p53-independent signaling in HCT116 cells. In another study, the ruthenium(II) N-heterocyclic carbene complex with naphthalimide led to apoptosis in CRC HCT116 cells by ROS-induced p38 MAPK pathway activation [24]. On the other hand, we observed that Ru/5-FU induces cell death by MAPK- and p53-independent pathways.

Ru/5-FU also regulated the autophagy markers LC3B and p62, which seems to be due to mTOR inhibition, a negative modulator of autophagy. Autophagy is a physiological process that presents dual action in cancer progression and can be associated with drug resistance and/or cell death [25–27]. Herein, we demonstrated an increase in Ru/5-FU-induced apoptosis in cells cotreated with autophagy inhibitors, indicating that Ru/5-FU-induced autophagy plays a cytoprotective role in HCT116 cells. Similarly, Ru(II) complexes combined with the β-carboline alkaloid when associated with autophagic inhibitors induced enhanced apoptosis in HeLa cells [28]. In this context, autophagic inhibitors such as chloroquine and hydroxychloroquine have been studied in clinical trials to overcome resistance and make tumor cells more sensitive to treatment [27, 29].

Ru/5-FU also suppressed stemness in HCT116 CRC cells, which seemed to be due to inhibition of Akt/mTOR and NF-κB signaling, two known CSC molecular targets. Interestingly, Ru(II)-p-cymene complexes of mesalazine derivatives were also able to suppress stemness in CRC HT-29 cells [30]. A Ru(II) triazine complex has been reported to eliminate CSCs in CRC HCT-116 CD44+ and breast cancer MCF-7 CD44+ cells [31].

In particular, the subpopulation of CSCs is involved in cell migration and invasion. In this study, we observed that Ru/5-FU was able to inhibit migration and invasion and regulate the EMT markers E-cadherin and vimentin. A ruthenium arene complex was able to suppress the migration and invasion of ovarian adenocarcinoma A2780 cells [32]. Ruthenium polypyridyl complexes also reduced cell invasion in lung cancer A549 cells [33].

The in vivo antitumor activity of Ru/5-FU was also investigated in C. B-17 SCID mice grafted with HCT116 cells. Interestingly, Ru/5-FU inhibited tumor growth and experimental lung metastases with tolerated toxicity, although it did not show high selectivity in vitro. Ru(II) complexes with piplartine (15 μmol/kg/day), as well as a Ru(II)-thymine complex (1 and 2 mg/kg/day), also reduced tumor development in HCT116-grafted mice [11, 12]. A ruthenium 1,10-phenanthroline-5,6-dione arene complex inhibited HCT116 proliferation in zebrafish embryos [34].

Altogether, these results indicate that Ru/5-FU is an anti-CRC chemotherapy drug candidate with the ability to eliminate stemness in CRC cells.

## Material and methods

### Ru/5-FU synthesis

Ru/5-FU was synthesized and analyzed as previously described [10]. All chemicals of comparable purity or reagent grade were used as received from Sigma‒Aldrich (Sigma‒Aldrich, St. Louis, MO, United States). Solvents were purified by standard methods. Ru/5-FU was dissolved in sterile dimethyl sulfoxide (DMSO, Synth, Diadema, SP, Brazil) at a 5 mg/mL stock solution for all experiments and diluted with culture medium at various concentrations.

### Cell culture

A panel of 24 cancer cell lines, four primary cancer cells, two noncancer cell lines, one primary noncancer cell and one mutant and its parental cell line was selected for this study, as detailed in Table S6. The cells were cultured according to the manufacturer’s instructions for each cell line or ATCC animal cell culture guidelines. To maintain exponential growth, all cell lines were cultured in flasks at 37°C in 5% CO_2_ and subcultured every 3-4 days. Adherent cells were collected using a 0.25% trypsin EDTA solution (Sigma‒Aldrich). To validate the use of mycoplasma-free cells, all cell lines were tested for mycoplasma using a mycoplasma stain kit (Sigma‒Aldrich). The trypan blue assay was used to assess cell viability in all experiments. At the start of the culture, more than 90% of the cells were viable.

### Alamar blue assay

Cell viability was quantified by the Alamar blue assay, as previously described [35]. Briefly, cells were seeded in 96-well plates (7 × 10^3^ cells/well for adherent cells or 3 × 10^4^ cells/well for suspended cells). After 24 h, the drug was added to each well and incubated for 72 h. DOX (Laboratory IMA S.A.I.C., Buenos Aires, Argentina) and 5-FU (Sigma‒Aldrich) were used as positive controls. Four (for cell lines) or 24 h (for primary culture) before the end of incubation, 20 μL of resazurin (30 μM) (Sigma‒Aldrich) was added to each well. Absorbance at 570 and 600 nm was measured using a SpectraMax 190 Microplate Reader (Molecular Devices, Sunnyvale, CA, USA).

### Gene expression analysis

HCT116 cells were incubated with 4 µM Ru/5-FU for 12 h, and total RNA was isolated using the RNeasy Plus Mini Kit (Qiagen; Hilden, Germany) according to the manufacturer’s instructions. The RNA was analyzed for purity and quantified by a NanoDrop® 1000 spectrophotometer (Thermo Fisher Scientific, Waltham, Massachusetts, USA), and a Superscript VILO™ Kit (Invitrogen Corporation; Waltham, MA, USA) was used for RNA reverse transcription. TaqMan® array human cancer drug targets 96-well plate, fast (ID RPRWENH, Applied Biosystems™, Foster City, CA, USA) was used for the gene expression study by qPCR using an ABI ViiA7 system (Applied Biosystems™). The cycle conditions comprised 2 min at 50 °C, 10 min at 95 °C, and then 40 cycles of 15 s at 95 °C and 1 min at 60 °C. All experiments were performed in DNase/RNase-free conditions. The relative quantification (RQ) of mRNA expression was calculated by the 2^-ΔΔCT^ method [36] using Gene Expression Suite™ Software (Applied Biosystems™), and cells treated with the negative control (0.2% DMSO) were used as a calibrator. The reactions were normalized by the geometric mean of the RQ of the reference genes GAPDH, B2M, UBC, PGK1, RPLP0 and TRFC. The genes were considered to be upregulated if RQ ≥ 2, which means that the gene expression in Ru/5-FU-treated cells was at least twice that of the negative control-treated cells. Similarly, the genes were considered to be downregulated if RQ ≤ 0.5, which means that the gene expression in Ru/5-FU-treated cells was at least half of that of the negative control-treated cells.

### Flow cytometry assays

Protein expression levels were quantified by flow cytometry using primary antibodies conjugated with specific fluorochromes as detailed in Table S7. For intracellular protein staining, cells were collected, fixed for 10 min in 0.5–1 mL of 4% formaldehyde at 37 °C and chilled on ice for 1 min. The cells were permeabilized by slowly adding ice-cold 100% methanol to prechilled cells while gently vortexing to a final concentration of 90% methanol and were incubated for 30 min on ice. After washing with incubation buffer (0.5% bovine serum albumin in PBS), antibodies were added and incubated for 1 h at room temperature. After washing with PBS, cell fluorescence was measured by flow cytometry.

For cell surface protein staining, cells were washed with incubation buffer (0.5% bovine serum albumin in PBS), and antibodies were added and incubated for 1 h at room temperature. Then, the cells were washed with PBS, and cell fluorescence was determined by flow cytometry. For quantification of CD133-positive cells, YO-PRO-1 (Sigma‒Aldrich) was used to select viable cells.

For the functional assay, cell viability was quantified using annexin V-FITC/PI (FITC Annexin V Apoptosis Detection Kit I, BD Biosciences, San Jose, CA, USA) or YO-PRO-1/propidium iodide (PI) (Sigma‒Aldrich). The following inhibitors were used: SP 600125 (JNK inhibitor, Cayman Chemical); PD 169316 (p38 MAPK inhibitor, Cayman Chemical); U-0126 (MEK inhibitor, Cayman Chemical); cyclic pifithrin-α (p53 inhibitor, Cayman Chemical); chloroquine (autophagy inhibitor, Ipca Laboratories, Mumbai, MH, India); 3-methyladenine (autophagy inhibitor, Sigma‒Aldrich); and lithium chloride (Wnt activator, Sigma‒Aldrich).

Internucleosomal DNA fragmentation and cell cycle distribution were analyzed by PI staining using a solution containing 0.1% Triton X-100, 2 µg/mL PI, 0.1% sodium citrate, and 100 µg/mL RNAse (all from Sigma‒Aldrich) and incubated in the dark for 15 min at room temperature [37]. Then, cellular fluorescence was evaluated by flow cytometry.

For all flow cytometry analyses, cell fluorescence was measured by a BD LSRFortessa cytometer using BD FACSDiva Software (BD Biosciences) and FlowJo Software 10 (FlowJo Lcc; Ashland, OR, USA). For intracellular staining, at least 10,000 events were evaluated per sample, while at least 30,000 events were acquired per sample for cell surface protein staining. Doublets were removed using FSC-A versus FCS-H and SCC-A versus SCC-H, according to Figure S20. Cellular debris was removed from cell acquisition.

### Phospho-specific ELISA

Histone H2AX (pS139) (catalog number #DYC2288-2), JNK2 (pT183/Y185) (catalog number #DYC2236-2), p38α (pT180/Y182) (catalog number #DYC869B-2) and ERK1 (pT202/Y204) (catalog number #DYC1825-2) were measured in cell lysates using sandwich ELISA kits (all from R&D Systems, Inc. Minneapolis, MN, USA) following the manufacturer’s instructions. Briefly, cells were harvested and suspended in lysis buffer containing a phosphatase and protease inhibitor cocktail and 1 mM PMSF (all from Sigma‒Aldrich). Total protein quantification was carried out in each sample by Pierce Assay (Thermo Fisher Scientific, Waltham, MA, USA) using BSA as a standard. Absorbance at 450 nm was acquired by a SpectraMax 190 Microplate Reader (Molecular Devices, Sunnyvale, CA, USA).

### Transmission electron microscopy analyses

For at least 2 h, the cells were fixed in 0.1 M sodium cacodylate buffer (pH 7.4) containing 2.5% glutaraldehyde and 2% paraformaldehyde. Following washing, the cells were treated for 1 h with 1% osmium tetroxide, 0.8% potassium ferricyanide, and 5 mM calcium chloride. Following another wash, the cells were dehydrated in an acetone series before being embedded in polybed epoxy resin. The ultrathin sections were stained with 2% aqueous uranyl acetate and 2% aqueous lead citrate before TEM analysis with a JEM-1230 microscope (JEOL, 1230, USA, Inc.).

### Immunofluorescence

HCT116 cells were plated onto coverslips placed in 24-well plates and treated with the drug for 24 h. Subsequently, the cells were washed twice with saline solution, permeabilized with Triton X-100 (0.5%), treated with RNAse (10 μg/mL), and incubated overnight with primary antibodies conjugated with a specific fluorochrome (see Table S7 for antibody details). On the following day, the cells were washed with PBS and mounted using Fluoromount-G with DAPI (Invitrogen, Thermo Fisher Scientific). The cells were analyzed with a Leica TCS SP8 confocal microscope (Leica Microsystems, Wetzlar, HE, Germany).

### Colony-forming assay

To check clonogenic ability, 1000 cells were seeded in 6-well plates with 6 mL of complete medium and treated with the drug for 24, 48 and 72 h incubation. Then, the medium was changed to drug-free fresh medium, and the cells were cultured for a total of 14 days. Cells were then fixed with methanol and stained with 0.5% crystal violet. The number of colonies of more than 50 cells was counted under an optical microscope (Nikon, TS100).

### Colonosphere assay

HCT116 cells were grown in serum-free DMEM-F12 supplemented with 20 ng/mL EGF (PeproTech, USA), 20 ng/mL bFGF (PeproTech, USA), and B27 supplement (Invitrogen, Carlsbad, CA, USA) at densities of 1.25 × 10^5^ cells/mL in 24-well low adhesion plates (Corning, USA). The cells were treated with 20, 10, 5, 2.5 and 1.25 µM. The cells were photographed after 0, 24, 48, and 72 h of incubation using an optical microscope (Leica, DMI8). In addition, cells were treated with 4 µM Ru/5-FU, stained with acridine orange (100 µg/ml) plus PI (2 µg/ml) and analyzed by confocal microscopy or stained with anti-CD133 antibody plus YO-PRO-1 and analyzed by flow cytometry.

### Wound healing assay

Wound healing assays were performed as previously described [38] with some modifications. Cells were grown to 80-90% confluency in 12-well plates, and a wound was made by dragging a plastic pipette tip across the surface of the cell. The remaining cells were washed three times in saline to remove cell debris, incubated with serum-free medium and treated with the drug. Migrating cells in front of the wound were photographed at 0 and 72 h of incubation using an optical microscope (Nikon, TS 100). The wound area was evaluated by ImageJ software (NIH, USA).

### Transwell migration assay

Cell invasion assays were carried out using Transwell plates as previously described [39]. First, cells were incubated in serum-free medium for 24 h. Cell culture inserts in 6-well plates (8 μm pore size; Corning, USA) were precoated with Matrigel (Corning, USA). A total of 10^6^ cells were suspended in 1.5 mL serum-free medium and added to the upper chamber, while 2 mL of medium containing 20% FBS was placed in the lower chamber. After 48 h of incubation, the cells that remained in the upper chamber were removed with cotton swabs. Cells on the lower surface of the membrane were fixed in 4% paraformaldehyde and stained with 0.5% crystal violet. The cells were photographed and counted using an optical microscope (Leica, DMI8).

### Animal models

A total of 66 C. B-17 SCID mice (female, 20-25 g) were supplied and housed under specific pathogen-free conditions by FIOCRUZ-BA animal facilities (Salvador, Bahia, Brazil) according to the experimental protocol that was approved by a local animal ethics committee (#10/2020). All mice were fed a standard pellet diet (with free access to food and water) and subjected to an artificially illuminated room (12 h dark/light cycle).

For the subcutaneous tumor implantation model, HCT116 cells (10^7^ cells/500 μL) were inoculated subcutaneously into the left front armpit of mice on day 0, as previously described [11, 12]. On the following day, the animals were treated by the intraperitoneal route (200 μL/animal) once a day for two weeks. Five groups of animals were randomized and formed: group 1 received vehicle (5% DMSO solution) (*n* = 10); group 2 received 5-FU at a dose of 15 mg/kg (*n* = 10); group 3 received DOX at a dose of 0.8 mg/kg (*n* = 10); group 4 received Ru/5-FU at a dose of 2 mg/kg (*n* = 10); and group 5 received Ru/5-FU at a dose of 4 mg/kg (*n* = 10). One day after treatment ended, the animals were euthanized with an anesthetic overdose (thiopental, 100 mg/kg), and tumors were excised, weighed and processed for histological analysis. The inhibition ratio (percent) was calculated as follows: inhibition ratio (percent) = [(*A* - *B*)/*A*] × 100, where A is the negative control’s average tumor weight and B is the treated group’s tumor weight.

For the experimental lung metastasis model, HCT116 cells (2 × 10^6^ cells/100 μL) were injected into the tail vein of each mouse on day 0. On the following day, the animals were treated by the intraperitoneal route (200 μL/animal) every other day for four weeks. Two groups of animals were randomized and formed: group 1 received vehicle (5% DMSO solution) (*n* = 8), and group 2 received Ru/5-FU at a dose of 4 mg/kg (*n* = 8). One day after treatment ended, the animals were euthanized with an anesthetic overdose (thiopental, 100 mg/kg), and the lungs were excised and fixed in 4% formaldehyde. The number of lung metastases per animal was counted and processed for histological analysis.

All animals were monitored for abnormalities throughout the experiment. Hematological analyses were carried out using an Advia 60 hematology system (Bayer, Leverkusen, Germany). The livers, kidneys, lungs, and hearts were removed, weighed, examined for color change, signs of gross lesion formation, and/or hemorrhaging and fixed in 4% formaldehyde. The organs were cut into pieces with a scalpel, dehydrated in a graded series of alcohol, washed in xylene and embedded in paraffin. The tissue was cut into 5 μm thick slices, stained with hematoxylin-eosin and/or periodic acid-Schiff (liver and kidney), and histologically examined under optical microscopy.

### Statistical analysis

The results are expressed as the mean of at least three repetitions (done in duplicate) ± S.E.M. or as IC_50_ values with 95% confidence intervals. For statistical analyses, the two-tailed unpaired Student’s *t* test was used to compare data with two groups, and one-way analysis of variance (ANOVA) followed by Dunnett’s multiple comparisons test was used to compare data with three or more groups by GraphPad Prism (Intuitive Software for Science; San Diego, CA, USA).

### Supplementary information

Supplemental material

## Data Availability

Data will be made available on request.
