# Supplementary material for: A novel ruthenium complex with 5-fluorouracil suppresses colorectal cancer stem cells by inhibiting Akt/mTOR signaling
Source: Cell Death Discov. 2023 Dec 16;9:460. doi: 10.1038/s41420-023-01759-6 (PMC10725484; doi:10.1038/s41420-023-01759-6)
Supplement: Supplementary file 1 — Supplemental material [file 41420_2023_1759_MOESM1_ESM.pdf]

Supplementary Material

A novel ruthenium complex with 5-fluorouracil suppresses colorectal cancer stem cells by inhibiting AKT/mTOR signaling

Valdenizia R. Silva[1], Luciano de S. Santos[1], Maria V. L. de Castro[1], Rosane B. Dias[1,2], Ludmila de F. Valverde[1], Clarissa A. G. Rocha[1,2], Milena B. P. Soares[1,3], Claudio A. Quadros[4,5], Rodrigo S. Correa[6], Alzir A. Batista[7], Daniel P. Bezerra[1,*]

[1]Gonçalo Moniz Institute, Oswaldo Cruz Foundation (IGM-FIOCRUZ/BA), Salvador, Bahia, 40296-710, Brazil.

[2]Department of Propedeutics, School of Dentistry of the Federal University of Bahia, Salvador, Bahia, 40110-909, Brazil.

[3]SENAI Institute of Innovation (ISI) in Health Advanced Systems, University Center SENAI/CIMATEC, Salvador, Bahia, 41650-010, Brazil.

[4]São Rafael Hospital, Rede D'Or/São Luiz, Salvador, Bahia, 41253-190, Brazil.

[5]Bahia State University, Salvador, Bahia, 41150-000, Brazil.

[6]Department of Chemistry, Federal University of Ouro Preto, Ouro Preto, Minas Gerais, 35400-000 Brazil.

[7]Department of Chemistry, Federal University of São Carlos, São Carlos, São Paulo, 13561-901 Brazil.

*Corresponding author: D. P. Bezerra, E-mail: daniel.bezerra@fiocruz.br Tel/Fax + 55 71 3176 2272.

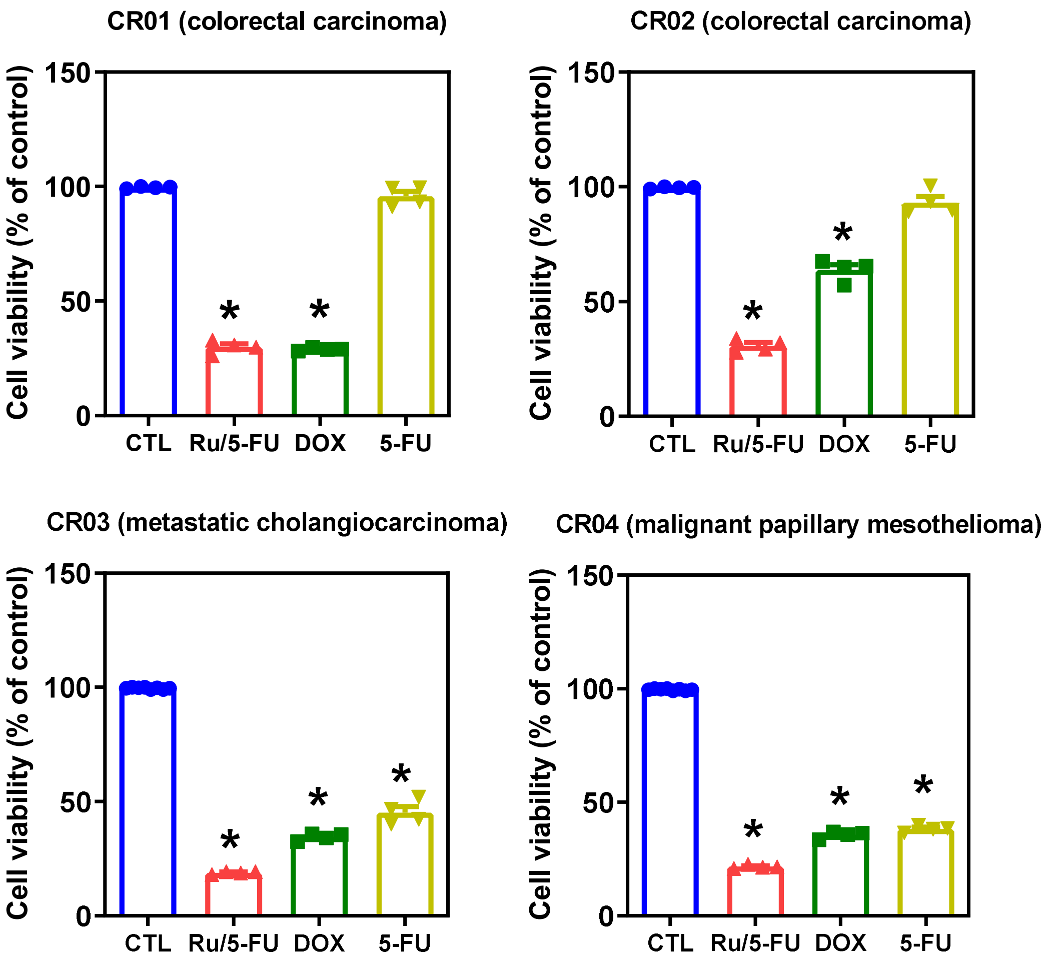

Figure S1. Effect of Ru/5-FU on primary cancer cells. Cells were exposed to 25 μg/mL of each compound (Ru/5-FU = 27.4 μM, DOX = 46 μM and 5-FU = 192.2 μM), and cell viability was quantified after 96 h of incubation using the Alamar blue assay. Data are shown as the mean ± S.E.M. of four replicates. * $P < 0.05$ compared to CTL by one-way ANOVA followed by Dunnett's multiple comparisons test.

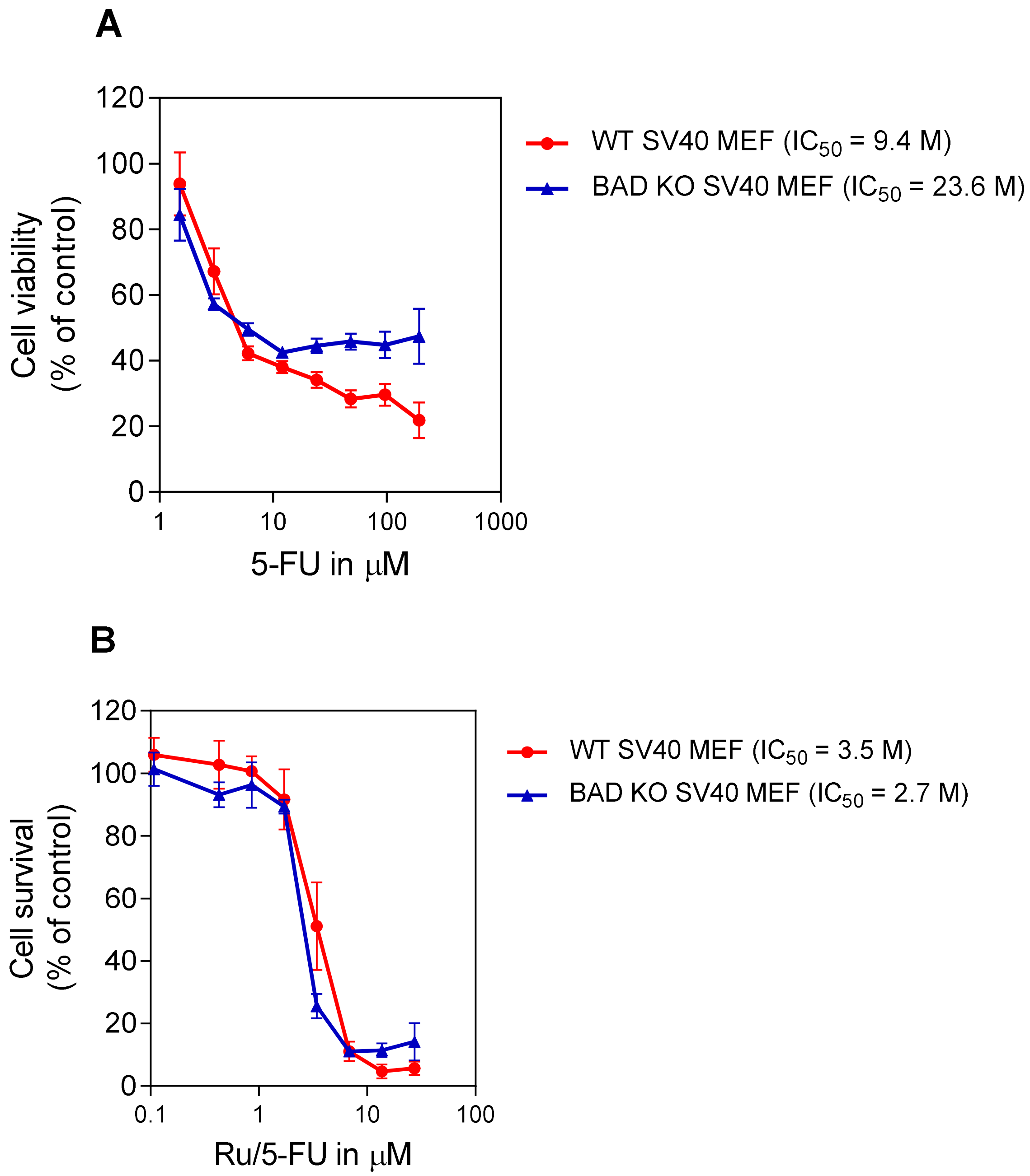

Figure S2. Survival curves of WT SV40 MEFs and BAD KO SV40 MEFs upon treatment with 5-FU (**A**) and Ru/5-FU (**B**). The curves were obtained from at least three independent experiments carried out in duplicate using the Alamar blue assay after 72 h of incubation. Data are shown as the mean ± S.E.M.

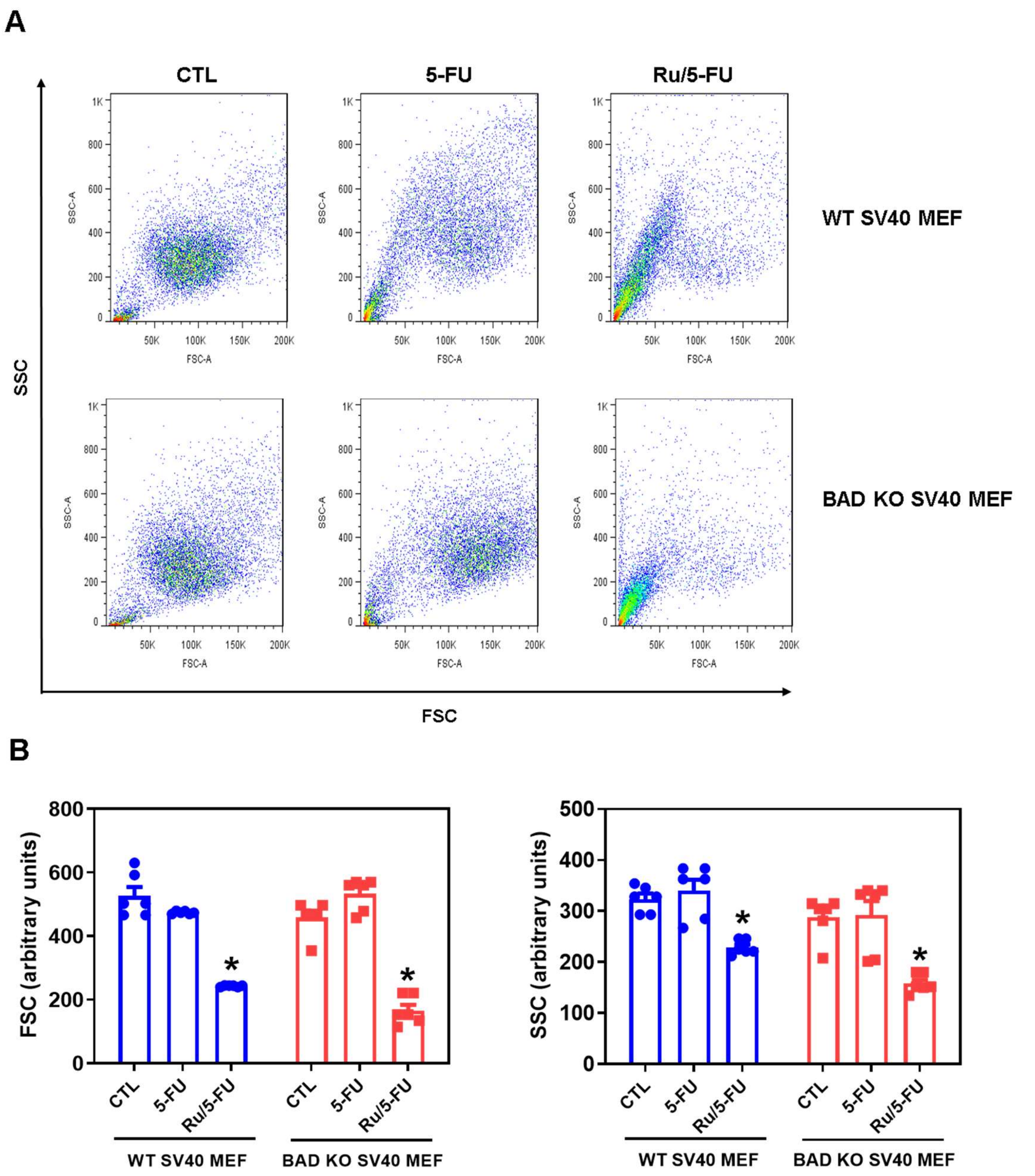

Figure S3. Light scattering features of WT SV40 MEFs and BAD KO SV40 MEFs after 48 h of incubation with 40 μM 5-FU and 4 μM Ru/5-FU. (**A**) Representative flow cytometry dot plots. (**B**) Quantification of forward scatter (FSC) and side scatter (SSC). The vehicle (0.2% DMSO) was used as a control (CTL). Data are shown as the mean ± S.E.M. of three independent experiments carried out in duplicate. * $P < 0.05$ compared to CTL by one-way ANOVA followed by Dunnett's multiple comparisons test.

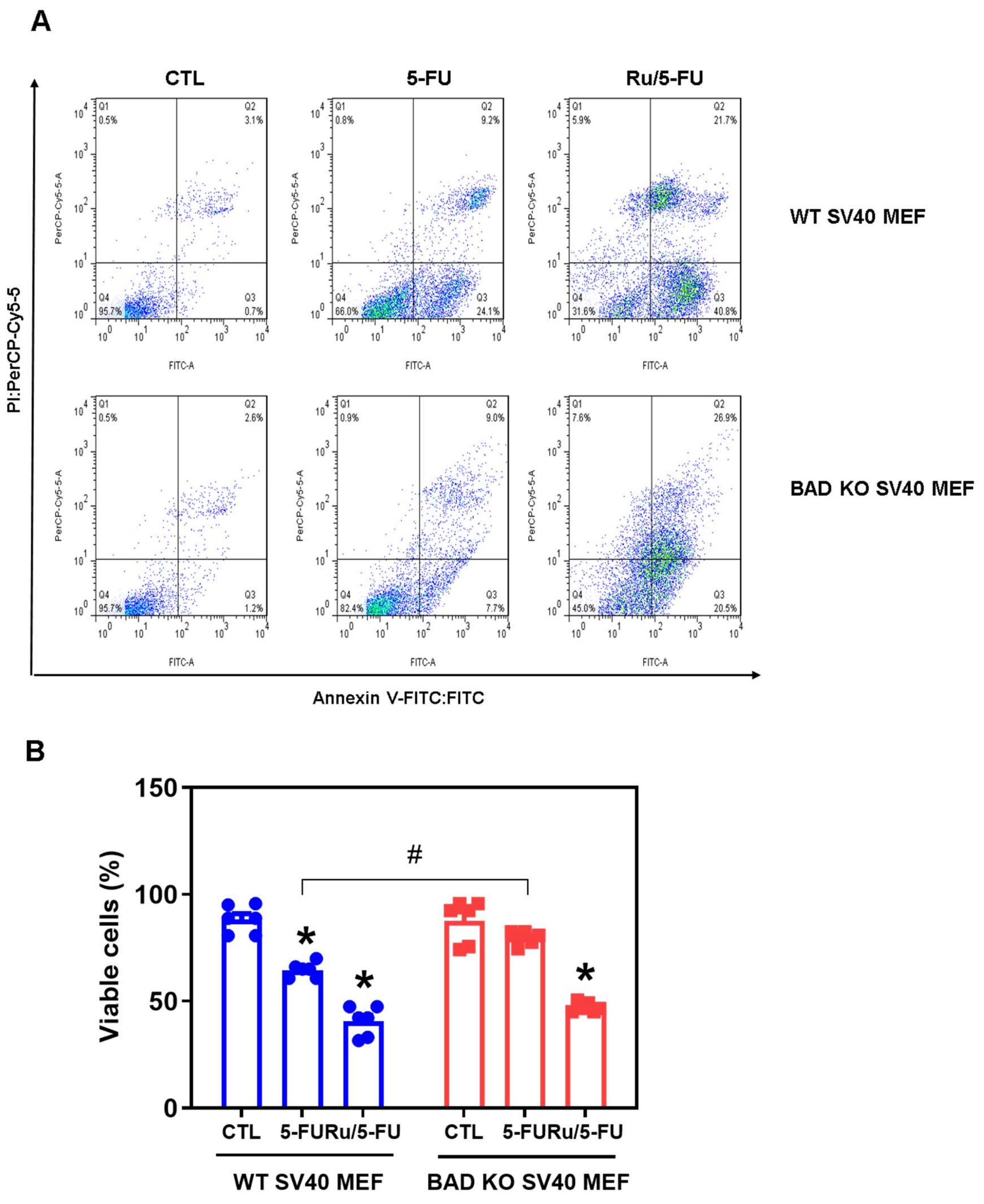

Figure S4. Induction of cell death in WT SV40 MEFs and BAD KO SV40 MEFs after 48 h of incubation with 40 µM 5-FU and 4 µM Ru/5-FU. (**A**) Representative flow cytometry dot plots. (**B**) Quantification of viable cells (annexin V-FITC/PI double-negative cells). The vehicle (0.2% DMSO) was used as a control (CTL). Data are shown as the mean ± S.E.M. of three independent experiments carried out in duplicate. * $P < 0.05$ compared to CTL by one-way ANOVA followed by Dunnett's multiple comparisons test. # $P < 0.05$ compared to the respective treatment in the wild-type cell line by Student's *t* test.

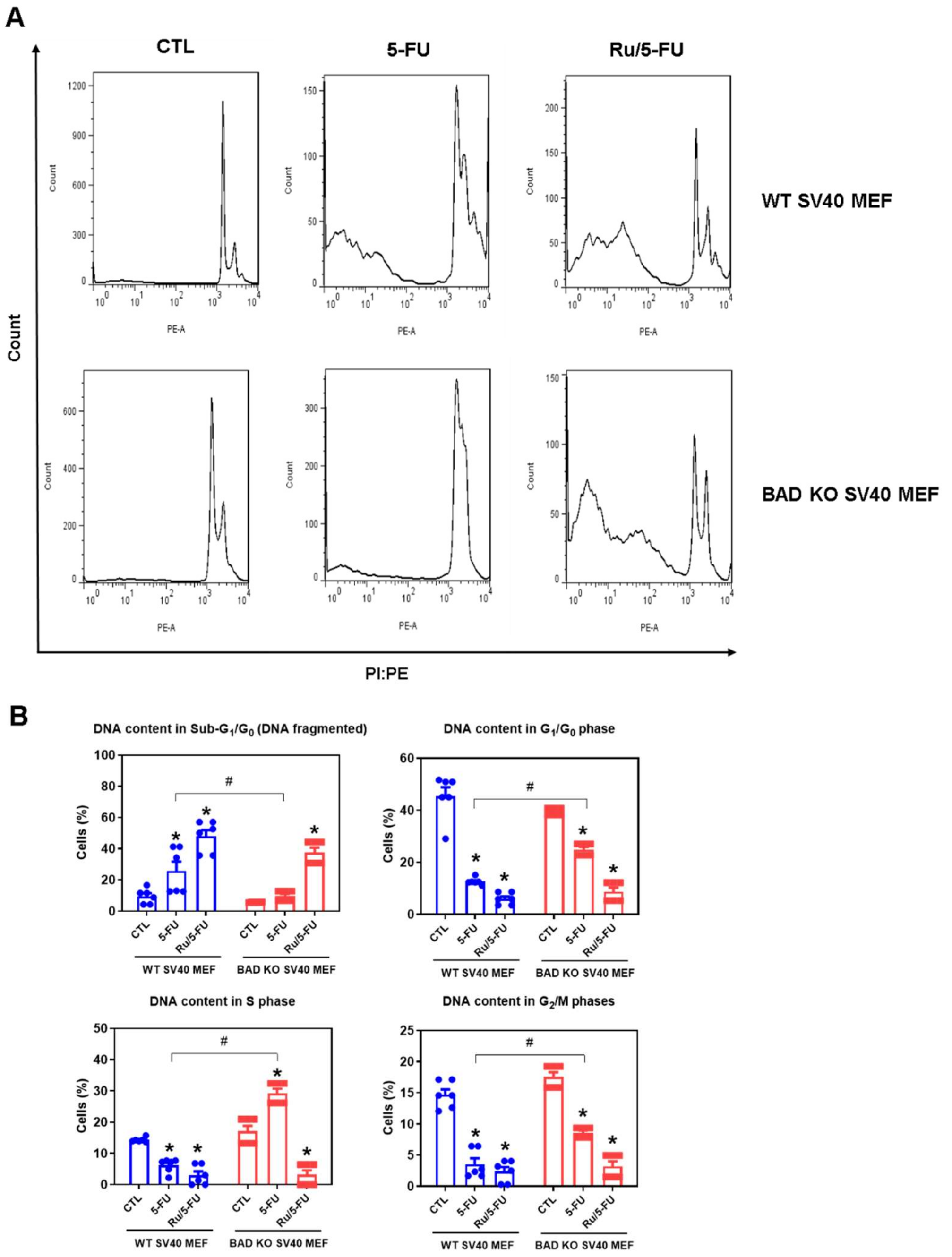

Figure S5. Cell cycle distribution of WT SV40 MEFs and BAD KO SV40 MEFs after 48 h of incubation with 40 μM 5-FU and 4 μM Ru/5-FU. (**A**) Representative flow cytometry histograms. (**B**) Quantification of cell cycle distribution. The vehicle (0.2% DMSO) was used as a control (CTL). Data are shown as the mean ± S.E.M. of three independent experiments carried out in duplicate. * $P < 0.05$ compared to CTL by one-way ANOVA followed by Dunnett's multiple comparisons test. # $P < 0.05$ compared to the respective treatment in the wild-type cell line by Student's *t* test.

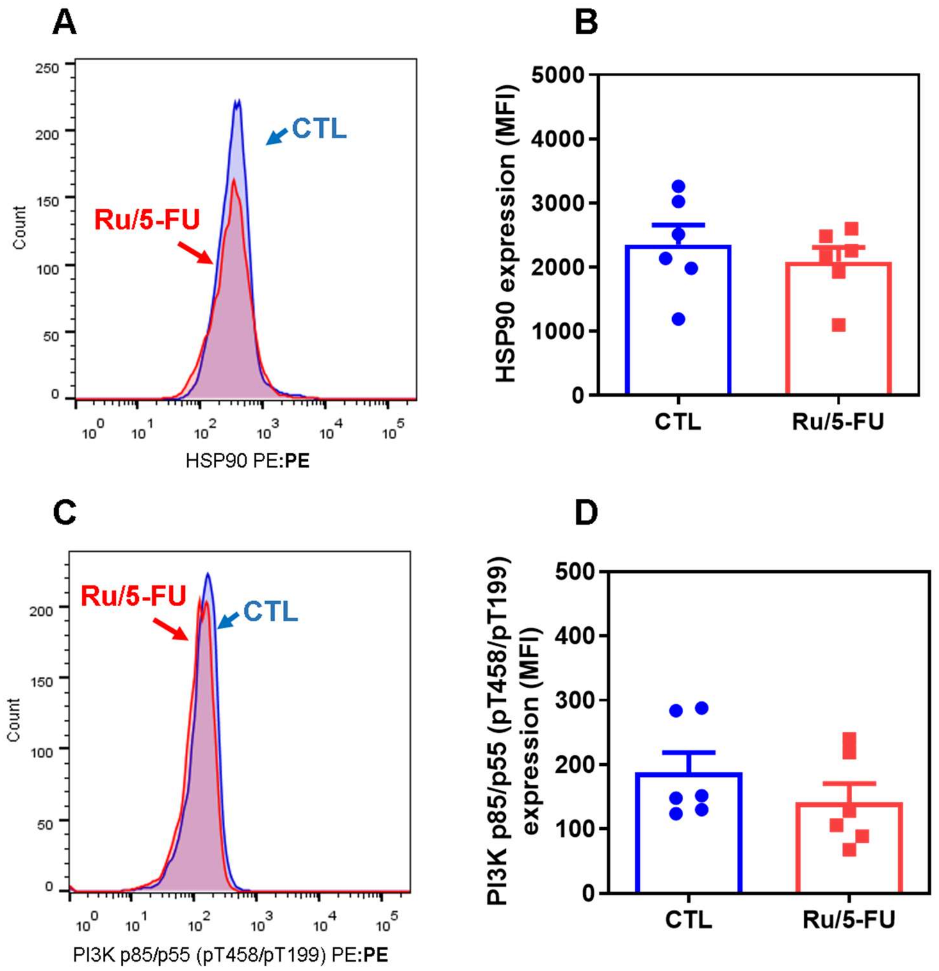

Figure S6. Quantification of HSP90 (**A**) and PI3K p85/p55 (pT458/pT199) (**B**) expression in HCT116 cells after 24 h of incubation with 4 µM Ru/5-FU, as determined by flow cytometric analysis. The vehicle (0.2% DMSO) was used as a control (CTL). Data are shown as the mean ± S.E.M. of three independent experiments carried out in duplicate. MFI: Mean fluorescence intensity.

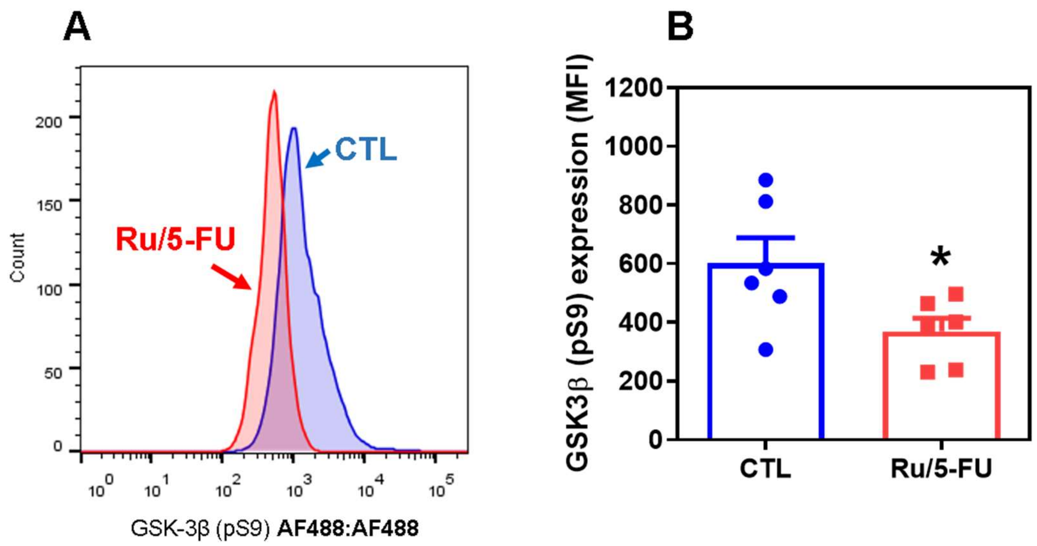

Figure S7. Quantification of GSK3 (pS9) expression in HCT116 cells after 24 h of incubation with 4 μM Ru/5-FU, as determined by flow cytometric analysis. The vehicle (0.2% DMSO) was used as a control (CTL). Data are shown as the mean ± S.E.M. of three independent experiments carried out in duplicate. * $P < 0.05$ compared to CTL by Student's *t* test. MFI: Mean fluorescence intensity.

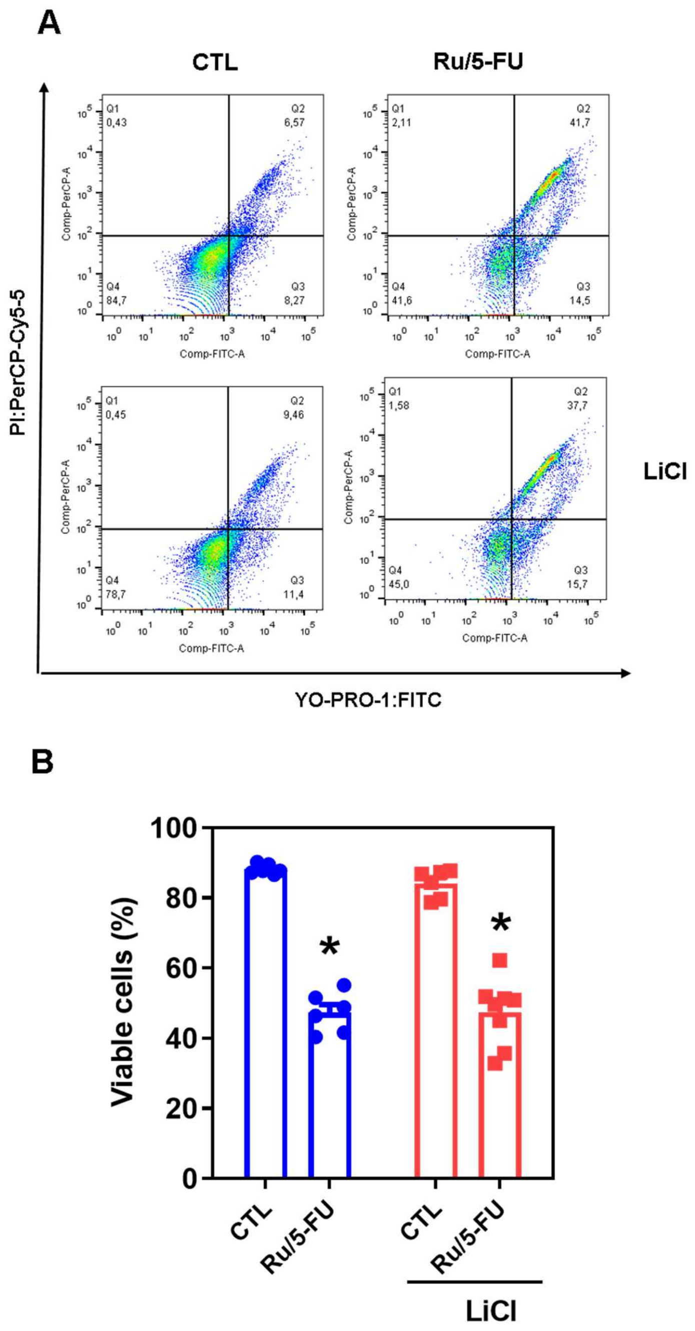

Figure S8. Action of lithium chloride (LiCl, a Wnt activator) on Ru/5-FU-induced HCT116 cell death. (**A**) Representative flow cytometry dot plots. (**B**) Quantification of viable HCT116 cells (YO-PRO-1/PI double-negative cells). The cells were pretreated with 25 mM LiCl and then incubated with 4 μM Ru/5-FU for 48 h. The vehicle (0.2% DMSO) was used as a control (CTL). Data are shown as the mean ± S.E.M. of three independent experiments carried out in duplicate. * $P < 0.05$ compared to CTL by Student's *t* test.

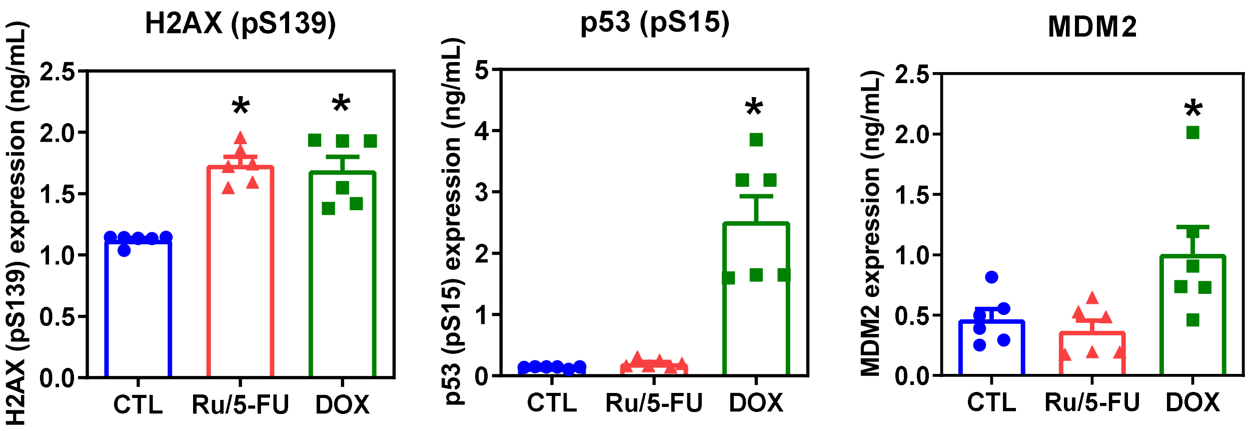

Figure S9. Quantification of histone H2AX (S139) (**A**), p53 (pS15) (**B**) and MDM2 (**C**) expression in HCT116 cells after 24 h of incubation with 4 μM Ru/5-FU, as determined by ELISA. Vehicle (0.2% DMSO) was used as a negative control (CTL), and DOX (1 μM) was used as a positive control. Data are shown as the mean ± S.E.M. of three independent experiments carried out in duplicate. * $P <$ 0.05 compared to CTL by one-way ANOVA followed by Dunnett's multiple comparisons test.

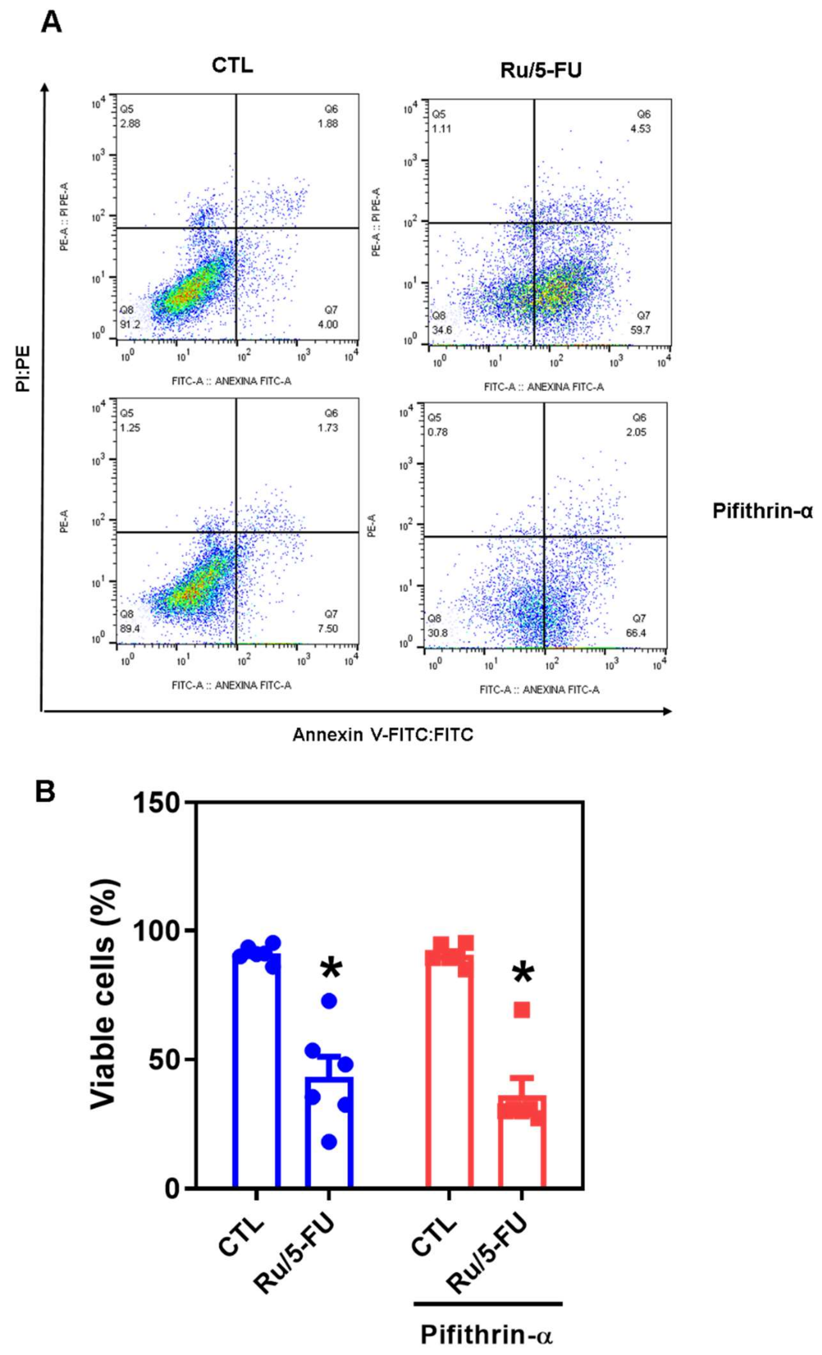

Figure S10. Effect of pifithrin-α (a p53 inhibitor) on Ru/5-FU-induced cell death in HCT116 cells. (**A**) Representative flow cytometric dot plots. (**B**) Quantification of viable HCT116 cells (annexin V-FITC/PI double-negative cells). The cells were pretreated with 10 µM pifithrin-α and then incubated with 4 µM Ru/5-FU for 48 h. The vehicle (0.2% DMSO) was used as a control (CTL). Data are shown as the mean ± S.E.M. of at least three independent experiments carried out in duplicate. * $P < 0.05$ compared to CTL by Student's *t* test.

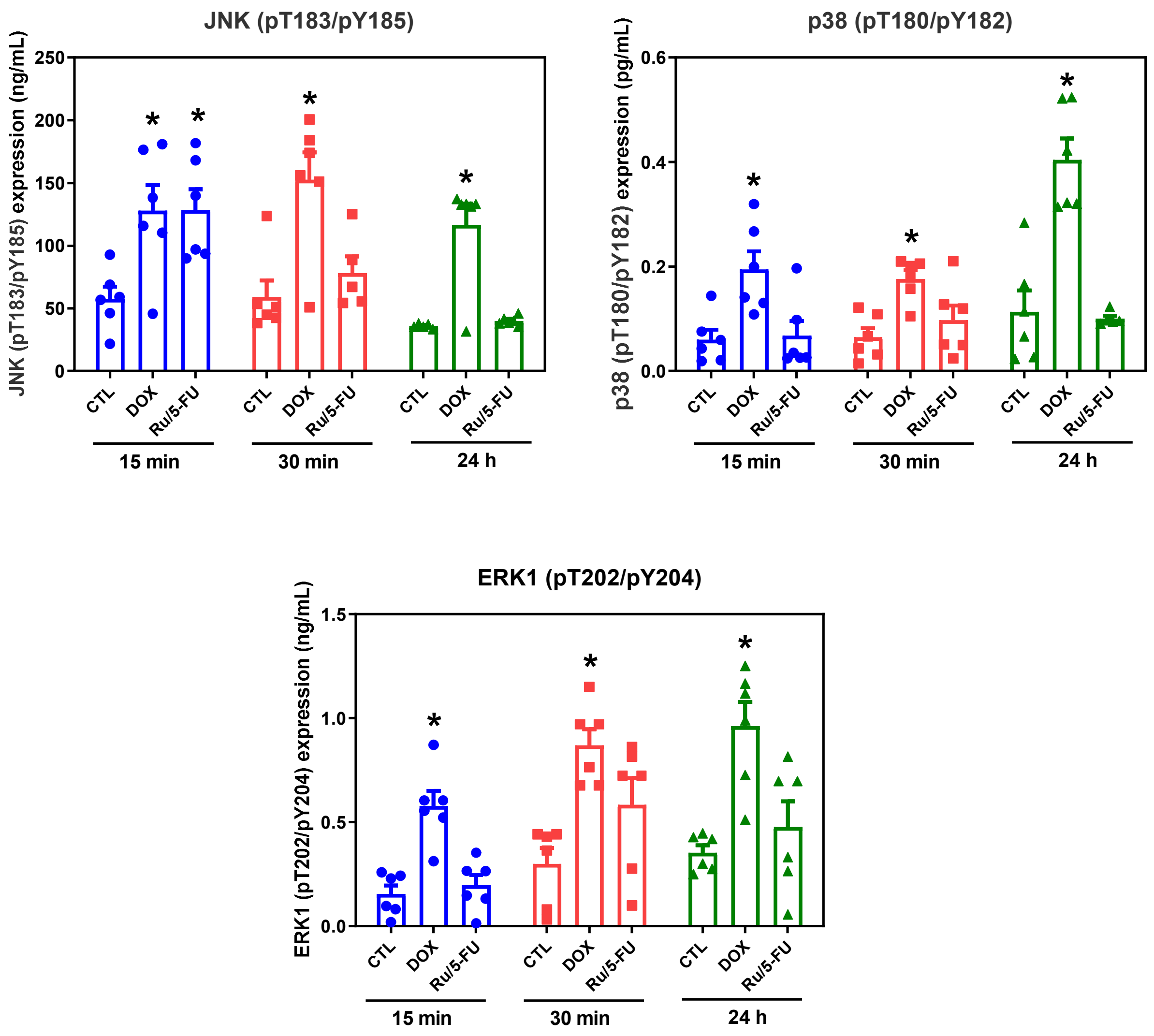

Figure S11. Quantification of JNK2 (pT183/pY185), p38α (pT180/pY182) and ERK1 (pT202/pY204) expression in HCT116 cells after 24 h of incubation with 4 μM Ru/5-FU, as determined by ELISA. Vehicle (0.2% DMSO) was used as a negative control (CTL), and DOX (1 μM) was used as a positive control. Data are shown as the mean ± S.E.M. of three independent experiments carried out in duplicate. * $P < 0.05$ compared to CTL by one-way ANOVA followed by Dunnett's multiple comparisons test.

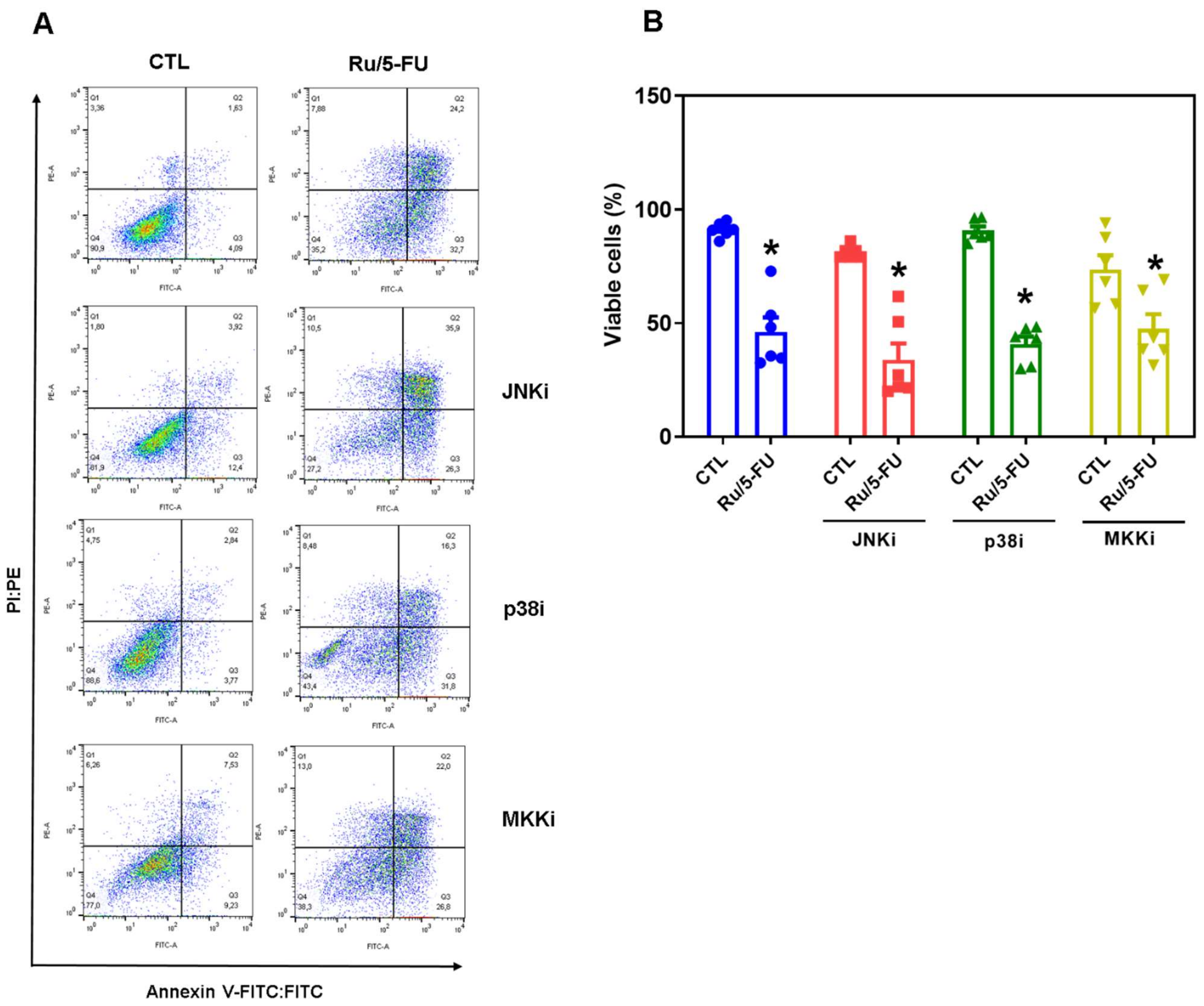

Figure S12. Action of SP 600125 (a JNKi), PD 169316 (a p38i) and U-0126 (a MKKi) on Ru/5-FU-induced cell death in HCT116 cells. (**A**) Representative flow cytometric dot plots. (**B**) Quantification of viable HCT116 cells (annexin V-FITC/PI double-negative cells). The cells were pretreated with 5 μM SP 600125, 5 μM PD 169316 or 5 μM U-0126 and then incubated with 4 μM Ru/5-FU for 48 h. The vehicle (0.2% DMSO) was used as a control (CTL). Data are shown as the mean ± S.E.M. of three independent experiments carried out in duplicate. * $P < 0.05$ compared to CTL by Student's *t* test.

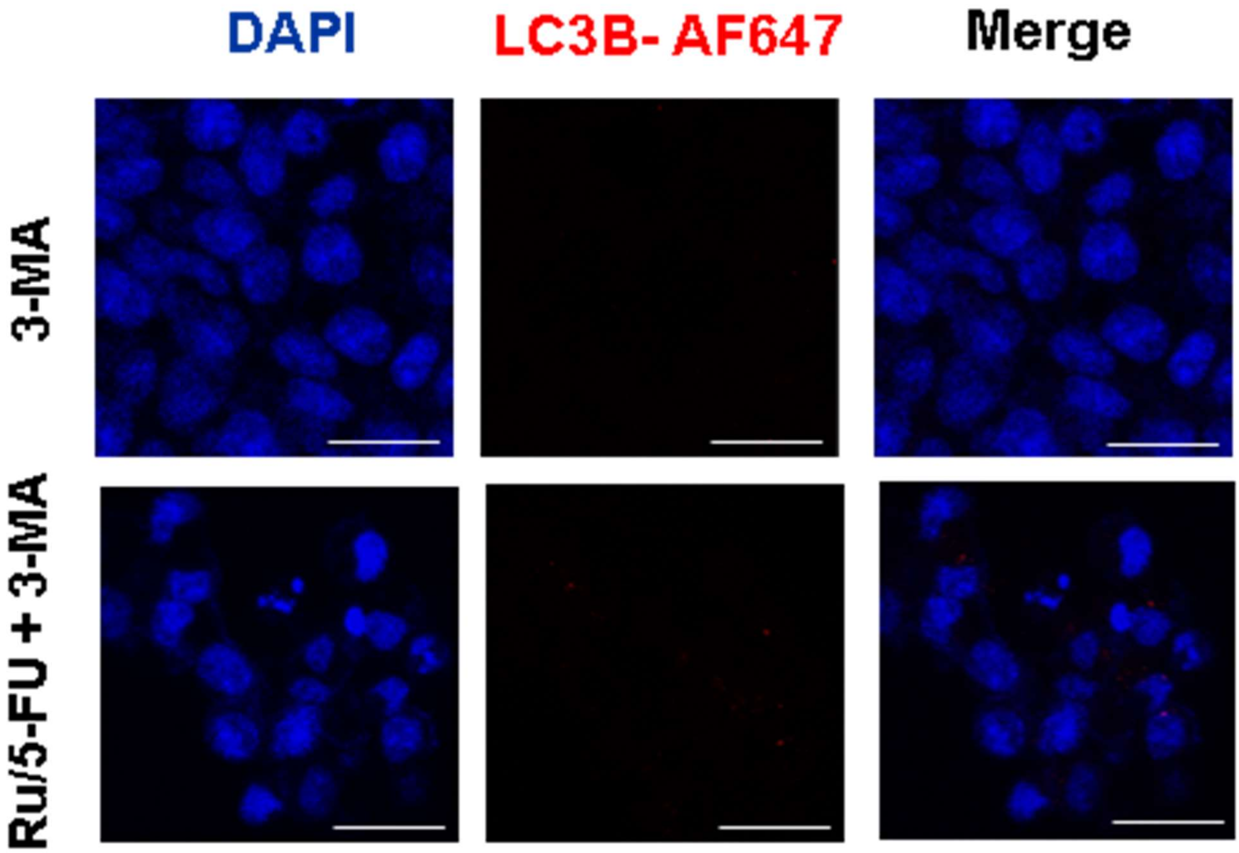

Figure S13. Representative immunofluorescence images of LC3B in HCT116 cells. The cells were pretreated with 5 mM 3-MA and then incubated with 4 μM Ru/5-FU for 24 h. The vehicle (0.2% DMSO) was used as a control (CTL). Scale bar = 25 μm.

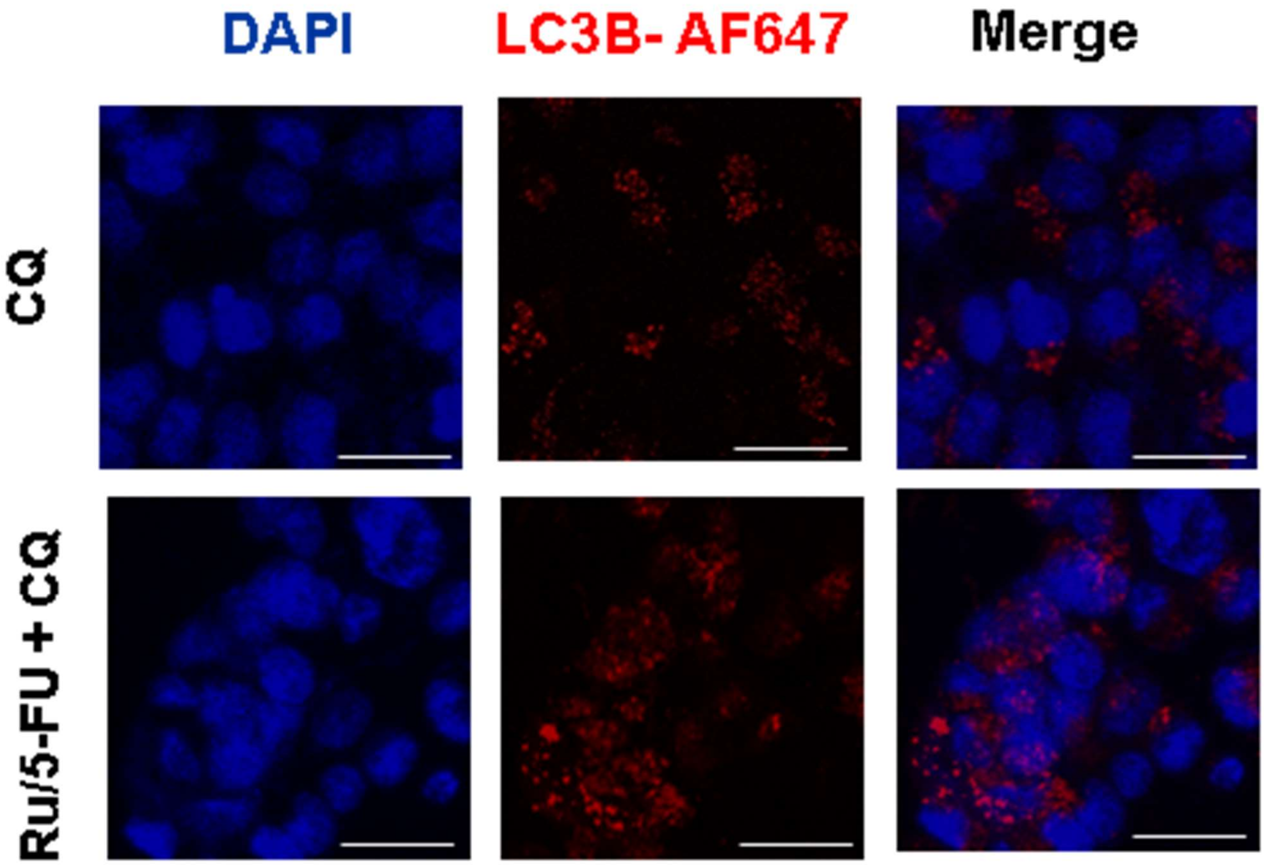

Figure S14. Representative immunofluorescence images of LC3B in HCT116 cells. The cells were pretreated with 50 μM CQ and then incubated with 4 μM Ru/5-FU for 24 h. The vehicle (0.2% DMSO) was used as a control (CTL). Scale bar = 25 μm.

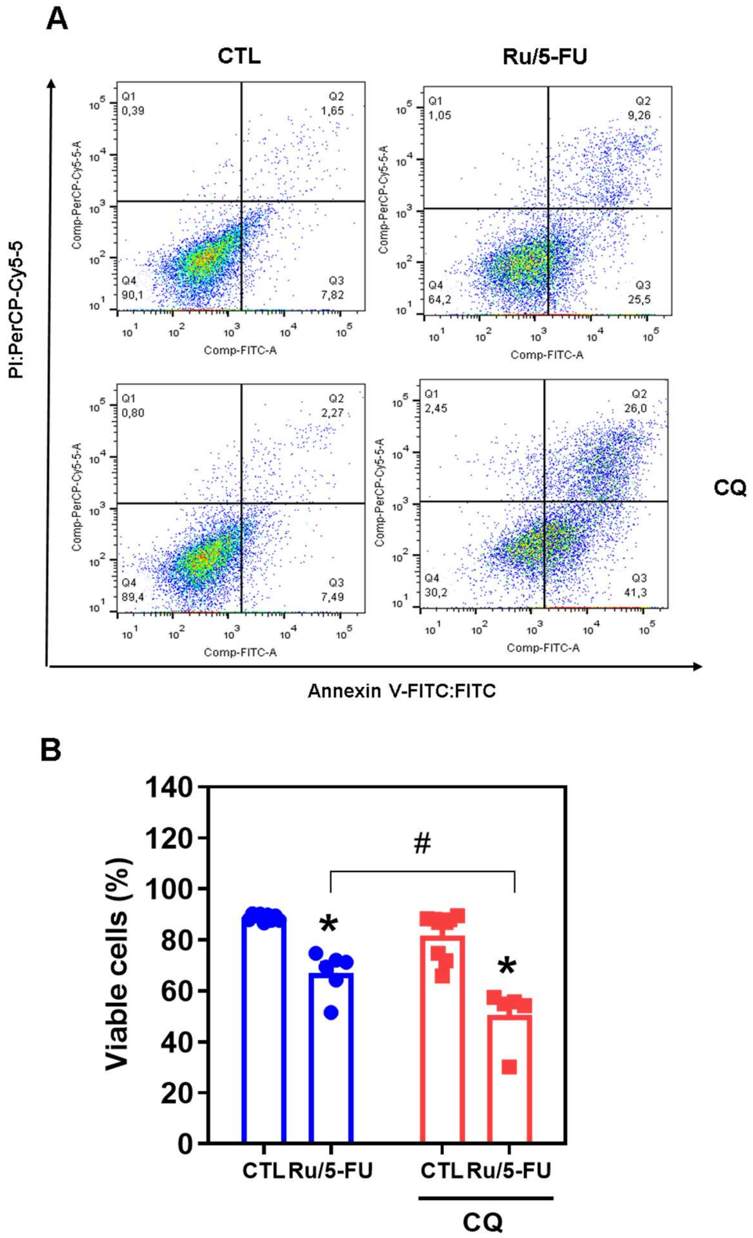

Figure S15. Effect of chloroquine (CQ, an autophagy inhibitor) on Ru/5-FU-induced cell death in HCT116 cells. (**A**) Representative flow cytometry dot plots. (**B**) Quantification of viable HCT116 cells (annexin V-FITC/PI double-negative cells). The cells were pretreated with 50 μM CQ and then incubated with 4 μM Ru/5-FU for 48 h. The vehicle (0.2% DMSO) was used as a control (CTL). Data are shown as the mean ± S.E.M. of three independent experiments carried out in duplicate. * $P < 0.05$ compared to CTL by Student's *t* test. # $P < 0.05$ compared to the respective treatment without inhibitor by Student's *t* test.

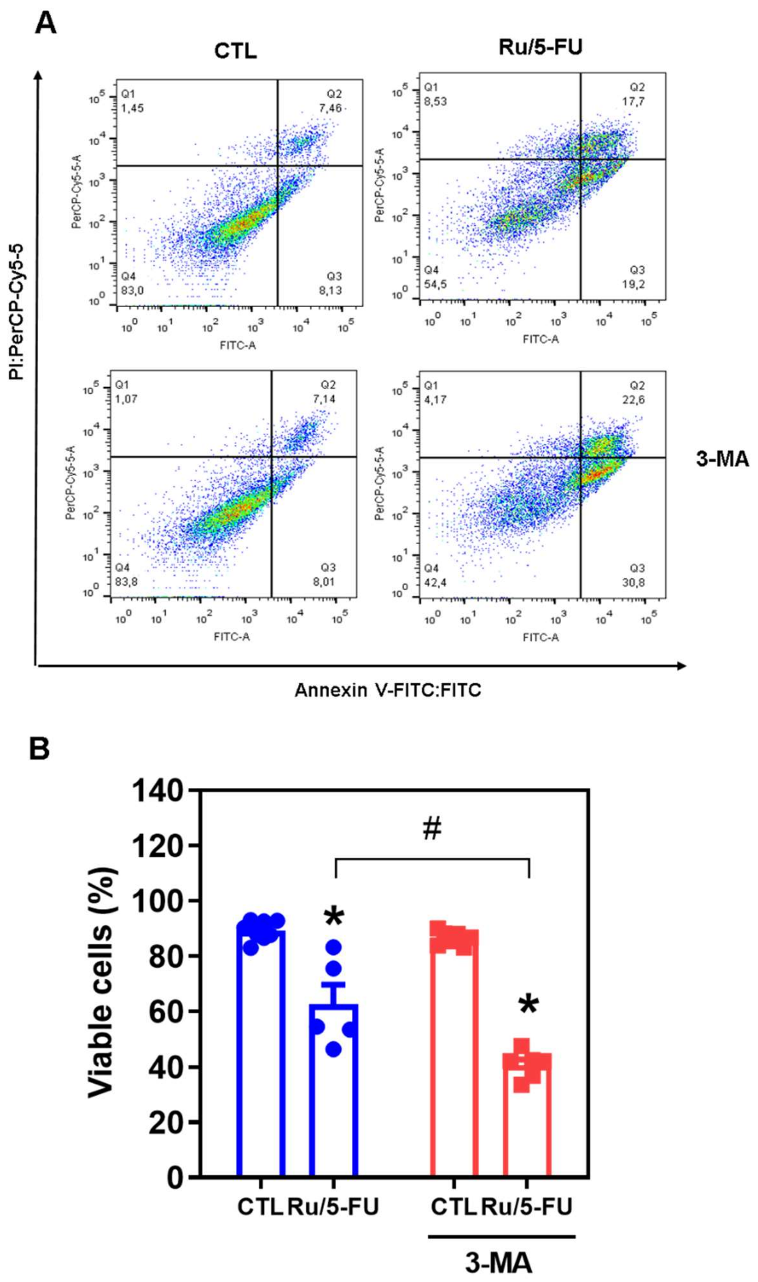

Figure S16. Effect of 3-methyladenine (3-MA, an autophagy inhibitor) on Ru/5-FU-induced cell death in HCT116 cells. (**A**) Representative flow cytometry dot plots. (**B**) Quantification of viable HCT116 cells (annexin V-FITC/PI double-negative cells). The cells were pretreated with 5 mM 3-MA and then incubated with 4 µM Ru/5-FU for 48 h. The vehicle (0.2% DMSO) was used as a control (CTL). Data are shown as the mean ± S.E.M. of three independent experiments carried out in duplicate. * $P < 0.05$ compared to CTL by Student's *t* test. # $P < 0.05$ compared to the respective treatment without inhibitor by Student's *t* test.

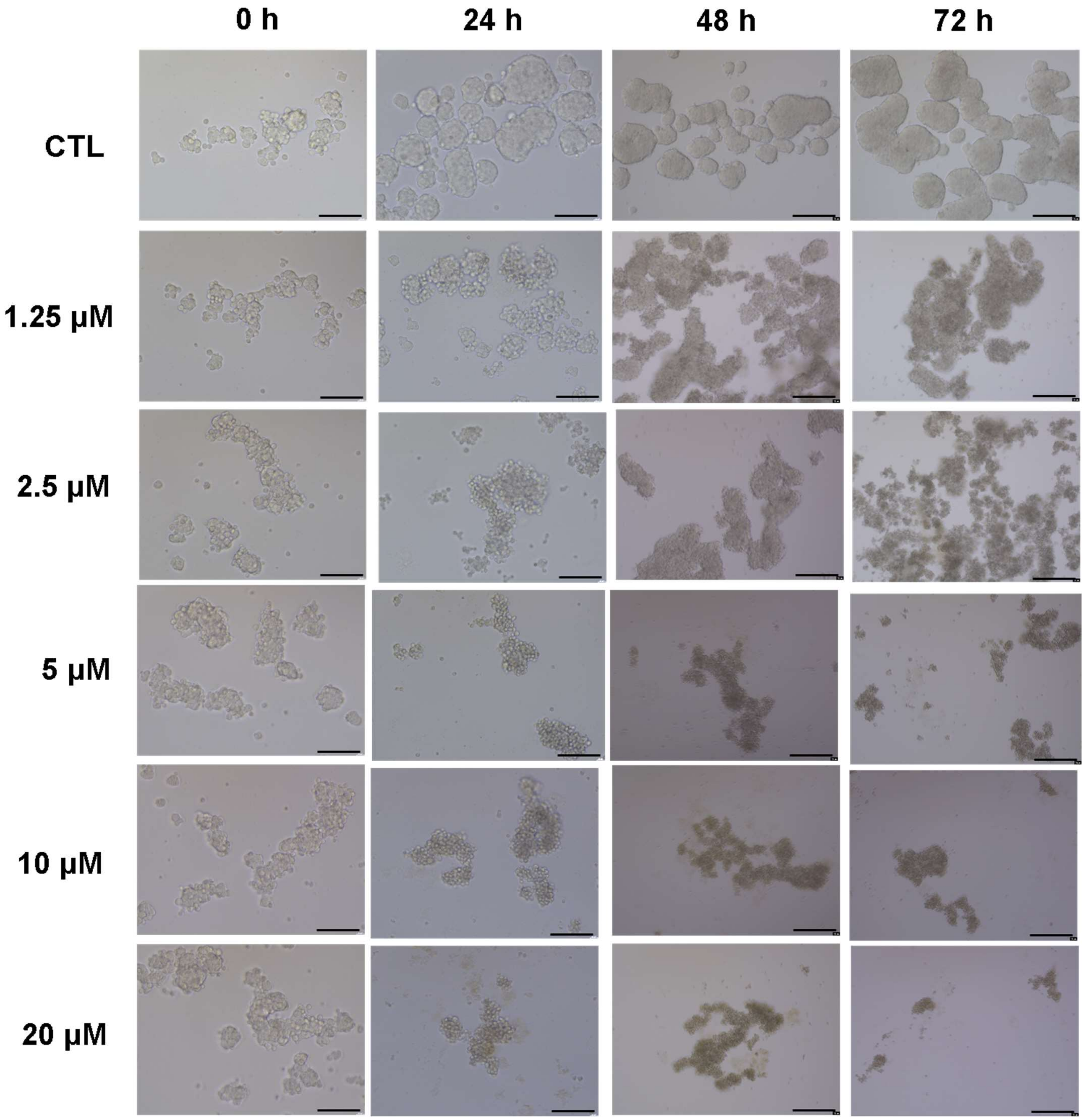

Figure S17. Representative images of colonospheres of HCT116 cells. The vehicle (0.2% DMSO) was used as a control (CTL). Scale bar = 100 μm.

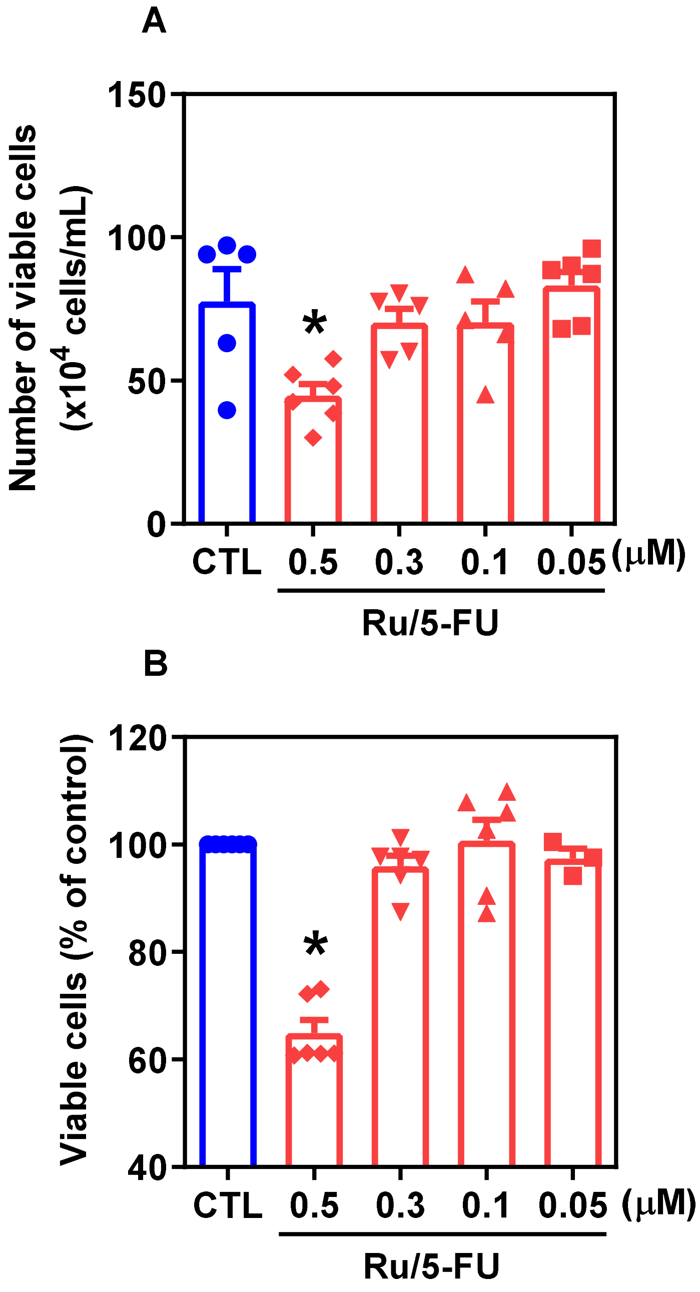

Figure S18. Quantification of viable HCT116 cells after 72 h of incubation with Ru/5-FU, as determined by trypan blue exclusion assay (**A**) and Alamar blue assay (**B**). The vehicle (0.2% DMSO) was used as a control (CTL). Data are shown as the mean ± S.E.M. of three independent experiments carried out in duplicate. * $P < 0.05$ compared to CTL by one-way ANOVA followed by Dunnett's multiple comparisons test.

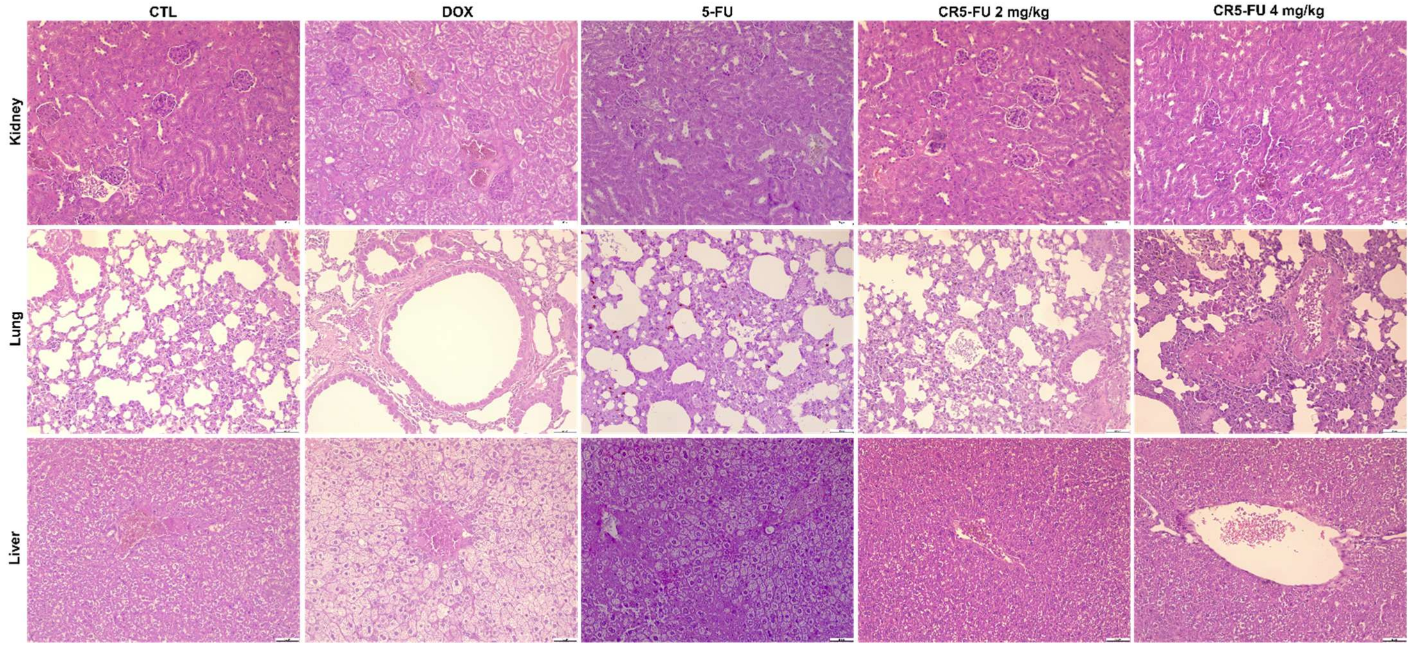

Figure S19. Representative histological images of the kidneys, lungs and livers of C. B-17 SCID mice inoculated with HCT116 cells. The tissues were stained with hematoxylin and eosin and analyzed by light microscopy. The negative control (CTL) was treated with the vehicle (5% DMSO) used to dilute Ru/5-FU. 5-FU (15 mg/kg) and DOX (0.8 mg/kg) were used as positive controls.

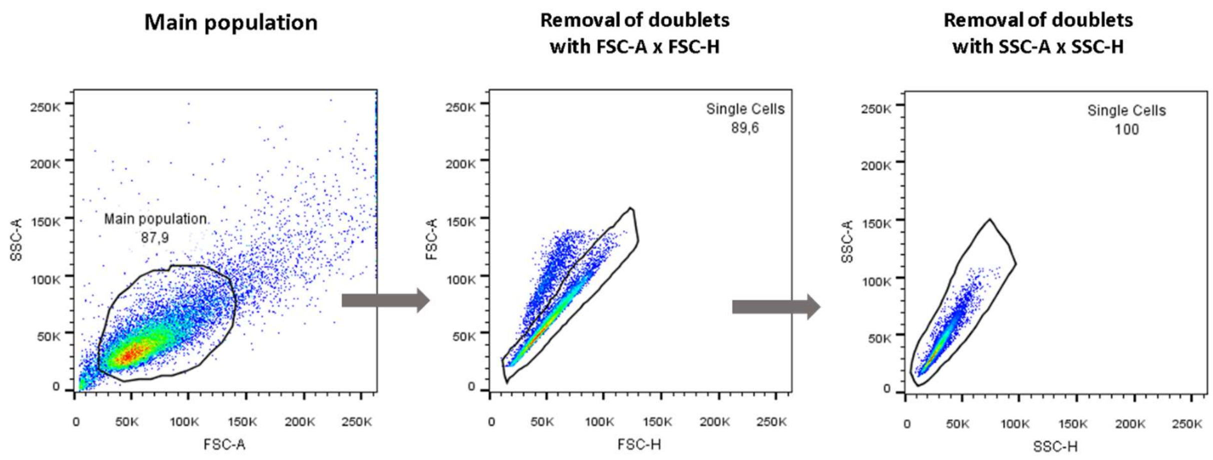

Figure S20. Representative gating strategy to remove doublets in flow cytometry analysis.

Table S1. IC_{50} values against cancer and noncancerous cells

Cells	IC_{50} and 95% CI (in µM)		
	**Ru/5-FU**	**DOX**	**5-FU**
Cancer cells			
HCT116	8.0	3.3	69.7
	5.9 – 10.9	2.6 – 4.6	49.4 – 98.3
HepG2	3.2	0.4	79.2
	2.7 – 3.8	0.3 – 0.55	62.4 – 100.3
NB4	4.3	0.1	13.8
	1.9 – 10.2	0.02 – 0.5	10.8 – 17.8
THP-1	2.9	0.1	5.2
	1.9 – 4.6	0.07 – 0.2	2.7 – 10.2
JUKART	4.3	0.2	9.3
	2.6 – 7.0	0.16 – 0.21	6.9 – 12.7
K-562	4.4	1.5	38.3
	2.0 – 9.5	0.5 – 4.5	26.9 – 54.2
HL-60	4.2	0.3	9.8
	3.4 – 5.2	0.27 – 0.43	7.6 – 12.5
KG-1a	5.4	0.4	6.9
	3.5 – 8.2	0.2 – 0.6	3.8 – 12.8
MDA-MB-231	2.7	0.6	N.d.
	1.9 – 3.6	0.2 – 1.3	
MCF-7	9.2	1.8	>192
	6.1 – 13.8	0.6 – 2.9	
4T1	3.7	2.2	15.6
	2.4 – 5.7	1.4 – 3.6	8.8 – 27.4
HSC-3	5.0	1.4	>192
	3.7 – 7.2	0.9 – 2.2	
CAL 27	5.3	0.2	37.4
	3.0 – 9.1	0.1 – 0.6	26.4 – 53.1
SCC-25	7.8	1.6	N.d.
	4.4 – 13.8	0.9 – 2.8	

SCC4	2.4<br>1.0 – 5.5	0.2<br>0.1 – 0.2	53.81<br>29.59 – 97.71
SCC-9	2.3<br>1.6 – 3.2	1.2<br>0.7 – 2.0	12.6<br>6.6 – 24.1
A549	7.5<br>5.7 – 9.8	2.4<br>2.0 – 2.8	130.7<br>107.6 – 158.7
NCI-H1299	4.03<br>3.11 – 5.22	0.72<br>0.55 – 1.15	Nd.
PANC-1	1.8<br>1.3 – 2.4	1.1<br>0.7 – 1.6	N.d
OVCAR-3	1.2<br>0.9 – 1.5	1.0<br>0.6 – 1.7	Nd.
DU 145	2.3<br>1.9 – 3.1	0.2<br>0.16 – 0.3	Nd.
U-87 MG	2.5<br>1.5 – 4.1	0.4<br>0.3 – 0.7	N.d.
A-375	1.7<br>1.3 – 2.2	0.2<br>0.1 – 0.2	21.9<br>14.8 – 32.3
B16-F10	3.2<br>2.1 – 5.0	0.5<br>0.4 – 0.7	Nd.
Noncancerous cells			
MRC-5	7.1<br>5.2 – 9.8	2.8<br>1.9 – 4.3	31.3<br>19.9 – 49.1
BJ	3.1<br>1.8 – 5.2	0.7<br>0.3 – 1.7	>192
PBMC	8.9<br>5.9 – 13.4	1.3<br>0.9 – 1.8	>192

These data were obtained by nonlinear regression from three independent experiments performed in duplicate after 72 h of incubation. Nd. Not determined.

Table S2. Selective index calculation

Cancer cells	Noncancer cells								
	MRC-5			BJ			PBMC		
	Ru/5-FU	DOX	5-FU	Ru/5-FU	DOX	5-FU	Ru/5-FU	DOX	5-FU
HCT116	0.89	0.85	0.45	0.38	0.21	Nd.	1.11	0.39	Nd.
HepG2	2.21	7	0.39	0.96	1.75	Nd.	2.87	3.25	Nd.
NB4	1.65	28	2.27	0.72	7	Nd.	2.07	13	Nd.
THP-1	2.44	28	6.01	1.60	7	Nd.	3.10	13	Nd.
JUKART	1.65	14.0	3.37	0.72	3.5	Nd.	2.07	6.5	Nd.
K-562	1.61	1.87	0.82	0.70	0.47	Nd.	2.02	0.87	Nd.
HL-60	1.69	9.3	3.20	0.74	2.3	Nd.	2.11	4.3	Nd.
KG-1a	1.32	7.0	4.53	0.57	1.75	Nd.	1.65	3.25	Nd.
MDA-MB-231	2.63	4.67	Nd.	1.15	1.17	Nd.	3.3	2.17	Nd.
MCF-7	0.77	1.55	Nd.	0.34	0.39	Nd.	0.96	0.72	Nd.
4T1	1.92	1.27	2.00	0.83	0.32	Nd.	2.41	0.59	Nd.
HSC-3	1.42	2.00	Nd.	0.62	0.5	Nd.	1.78	0.93	Nd.
CAL 27	1.34	14.0	0.84	0.58	3.50	Nd.	1.68	6.50	Nd.
SCC-25	0.91	1.75	N.d.	0.39	0.43	Nd.	1.14	0.81	Nd.
SCC4	2.96	14.0	0.58	1.29	3.50	Nd.	3.70	6.50	Nd.
SCC-9	3.08	2.33	2.48	1.35	0.58	Nd.	3.87	1.08	Nd.
A549	0.95	1.16	0.24	0.41	0.29	Nd.	1.19	0.54	Nd.
NCI-H1299	1.8	3.9	Nd.	0.8	1.0	Nd.	2.2	1.8	Nd.
PANC-1	3.94	2.54	Nd.	1.72	0.64	Nd.	4.94	1.18	Nd.
OVCAR-3	5.92	2.80	Nd.	2.58	0.70	Nd.	7.42	1.30	Nd.
DU 145	3.08	14.0	Nd.	1.34	3.50	Nd.	3.86	6.65	Nd.
U-87 MG	2.84	7.00	N.d.	1.24	1.75	Nd.	3.56	3.25	Nd.
A-375	4.18	14.0	1.43	1.82	3.50	Nd.	5.23	6.50	Nd.
B16-F10	2.22	5.60	Nd.	0.97	1.40	Nd.	2.78	2.60	Nd.

Data were calculated using the following formula: IS = IC_{50} [noncancer cells]/IC_{50} [cancer cells]. Nd. Not determined.

Table S3. The effect of Ru/5-FU on gene expression in HCT116 cells

Function/Assay ID	Gene symbol	Full name	RQ	
			CTL	Ru/5-FU
**Apoptosis**				
Hs00608023_m1	BCL2	BCL2, apoptosis regulator	1.0	0.903
Hs04194392_s1	BIRC5	baculoviral IAP repeat containing 5	1.0	0.463
**PI3 Kinases & Phosphatases**				
Hs00234508_m1	MTOR	mechanistic target of rapamycin	1.0	0.638
Hs00904054_m1	PIK3C2A	phosphatidylinositol-4-phosphate 3-kinase catalytic subunit type 2 alpha	1.0	1.155
Hs00176908_m1	PIK3C3	phosphatidylinositol 3-kinase catalytic subunit type 3	1.0	1.266
Hs00907957_m1	PIK3CA	phosphatidylinositol-4,5-bisphosphate 3-kinase catalytic subunit alpha	1.0	1.053
**Growth Factors & Receptors**				
Hs01076090_m1	EGFR	epidermal growth factor receptor	1.0	1.292
Hs01001580_m1	ERBB2	erb-b2 receptor tyrosine kinase 2	N.d.	N.d.
Hs00176538_m1	ERBB3	erb-b2 receptor tyrosine kinase 3	1.0	0.962
Hs01128657_m1	FIGF	c-fos induced growth factor	N.d.	N.d.
Hs01052961_m1	FLT1	fms related tyrosine kinase 1	N.d.	N.d.
Hs01047677_m1	FLT4	fms related tyrosine kinase 4	N.d.	N.d.
Hs01547656_m1	IGF1	insulin like growth factor 1	N.d.	N.d.
Hs00609566_m1	IGF1R	insulin like growth factor 1 receptor	1.0	0.921
Hs04188276_m1	IGF2	insulin like growth factor 2		
Hs00911700_m1	KDR	kinase insert domain receptor	1.0	1.723
Hs00174029_m1	KIT	KIT proto-oncogene receptor tyrosine kinase	N.d.	N.d.

Hs00998018_m1	PDGFRA	platelet derived growth factor receptor alpha	N.d.	N.d.
Hs01019589_m1	PDGFRB	platelet derived growth factor receptor beta	1.0	1.260
**Drug Metabolism**				
Hs01561483_m1	ABCC1	ATP binding cassette subfamily C member 1	1.0	0.692
Hs00943350_g1	GSTP1	glutathione S-transferase pi 1	1.0	0.123
Hs00153133_m1	PTGS2	prostaglandin-endoperoxide synthase 2	1.0	5.611
Hs01555214_g1	TXN	thioredoxin	1.0	0.950
Hs00917067_m1	TXNRD1	thioredoxin reductase 1	1.0	1.889
**G-Protein Signaling**				
Hs00357608_m1	RHOA	ras homolog family member A	1.0	0.758
Hs03676562_s1	RHOB	ras homolog family member B	1.0	0.808
**Hormone Receptors**				
Hs01046816_m1	ESR1	estrogen receptor 1	1.0	4.656
Hs01100353_m1	ESR2	estrogen receptor 2	1.0	1.325
Hs01556702_m1	PGR	progesterone receptor	N.d.	N.d.
**Heat Shock Proteins**				
Hs00743767_sH	HSP90AA1	heat shock protein 90 alpha family class A member 1	1.0	0.973
Hs00427665_g1	HSP90B1	heat shock protein 90 beta family member 1	1.0	1.012
**Receptor Tyrosine Kinase Signaling**				
Hs00178289_m1	AKT1	AKT serine/threonine kinase 1	1.0	0.392
Hs01086099_m1	AKT2	AKT serine/threonine kinase 2	1.0	0.516
Hs00157817_m1	GRB2	growth factor receptor bound protein 2	1.0	1.194
**Cathepsins**				
Hs00947439_m1	CTSB	cathepsin B	1.0	0.786
Hs00157205_m1	CTSD	cathepsin D	1.0	0.802
Hs00964650_m1	CTSL	cathepsin L	1.0	1.705

Hs00175407_m1	CTSS	cathepsin S	1.0	0.839
**Cell Cycle**				
Hs00947994_m1	CDC25A	cell division cycle 25A	1.0	0.662
Hs00938777_m1	CDK1	cyclin dependent kinase 1	1.0	0.736
Hs01548894_m1	CDK2	cyclin dependent kinase 2	1.0	0.728
Hs00364847_m1	CDK4	cyclin dependent kinase 4	1.0	0.731
Hs00358991_g1	CDK5	cyclin dependent kinase 5	1.0	0.328
Hs00361486_m1	CDK7	cyclin dependent kinase 7	1.0	2.236
Hs00992501_g1	CDK8	cyclin dependent kinase 8	1.0	0.793
Hs00977896_g1	CDK9	cyclin dependent kinase 9	1.0	1.007
Hs00540450_s1	MDM2	MDM2 proto-oncogene	1.0	2.150
Hs00967238_m1	MDM4	MDM4, p53 regulator	1.0	1.932
Hs00972650_m1	TERT	telomerase reverse transcriptase	1.0	0.277
**Topoisomerases, Type II**				
Hs01032137_m1	TOP2A	topoisomerase (DNA) II alpha	1.0	0.584
Hs00172259_m1	TOP2B	topoisomerase (DNA) II beta	1.0	1.475
**Transcription Factors**				
Hs01095345_m1	ATF2	activating transcription factor 2	1.0	0.661
Hs00153153_m1	HIF1A	hypoxia inducible factor 1 alpha subunit	1.0	0.537
Hs00158114_m1	IRF5	interferon regulatory factor 5	1.0	0.734
Hs00765730_m1	NFKB1	nuclear factor kappa B subunit 1	1.0	1.078
Hs01034249_m1	TP53	tumor protein p53	1.0	0.980
**Protein Kinases**				
Hs01582072_m1	AURKA	aurora kinase A	1.0	0.331
Hs00945858_g1	AURKB	aurora kinase B	1.0	0.549
Hs00152930_m1	AURKC	aurora kinase C	1.0	0.915
Hs00983227_m1	PLK1	polo like kinase 1	1.0	0.545
Hs00198320_m1	PLK2	polo like kinase 2	1.0	0.823
Hs00177725_m1	PLK3	polo like kinase 3	1.0	2.728
Hs00179514_m1	PLK4	polo like kinase 4	1.0	1.768
Hs00925200_m1	PRKCA	protein kinase C alpha	1.0	1.054
Hs00176998_m1	PRKCB	protein kinase C beta	N.d.	N.d.

Hs01090047_m1	PRKCD	protein kinase C delta	1.0	1.747
Hs00942886_m1	PRKCE	protein kinase C epsilon	1.0	1.523
**RAS Signaling**				
Hs00978050_g1	HRAS	HRas proto-oncogene, GTPase	1.0	0.999
Hs00364284_g1	KRAS	KRas proto-oncogene, GTPase	1.0	1.199
Hs00180035_m1	NRAS	NRas proto-oncogene, GTPase	1.0	0.820
**Histone Deacetylases**				
Hs00978031_g1	HDAC1	histone deacetylase 1	1.0	0.589
Hs00978031_g1	HDAC11	histone deacetylase 11	1.0	0.778
Hs00231032_m1	HDAC2	histone deacetylase 2	1.0	0.744
Hs00187320_m1	HDAC3	histone deacetylase 3	1.0	0.769
Hs01041648_m1	HDAC4	histone deacetylase 4	1.0	0.905
Hs00997427_m1	HDAC6	histone deacetylase 6	1.0	0.982
Hs01045864_m1	HDAC7	histone deacetylase 7	1.0	1.080
Hs00954353_g1	HDAC8	histone deacetylase 8	1.0	0.944
**Poly ADP-Ribose Polymerases**				
Hs00242302_m1	PARP1	poly(ADP-ribose) polymerase 1	1.0	0.516
Hs00173105_m1	PARP4	poly(ADP-ribose) polymerase family member 4	1.0	1.004
Hs00186671_m1	TNKS	tankyrase	1.0	2.856
**Structural Proteins**				
Hs00362403_g1	NTN3	netrin 3	N.d.	N.d.

HCT116 cells were treated with 4 μM Ru/5-FU for 12 h. The negative control (CTL) was treated with the vehicle (0.2% DMSO) used for diluting the compound tested. After treatment, total RNA was isolated and reverse transcribed. Gene expression was detected using a TaqMan® Array Human Cancer Drug Targets 96-well plate. The GAPDH, B2M, UBC, PGK1, RPLP0 and TRFC genes were used as endogenous genes for normalization. Values represent the relative quantitation (RQ) compared with the calibrator (cells treated with the negative control). The genes were considered to be upregulated if RQ ≥ 2 and downregulated if RQ ≤ 0.5. N.d. Not determined.

Table S4. Effect of Ru/5-FU treatment on body weight and relative organ weight of C. B-17 SCID mice inoculated with HCT116 cells

**Parameters**	**CTL**	**5-FU**	**DOX**	**Ru/5-FU**	**Ru/5-FU**
Dose (mg/kg)	-	15	0.8	2	4
Survival	10/10	5/10	10/10	10/10	9/10
Initial body weight (g)	22.7 ± 0.3	24.7 ± 0.6	24.5 ± 0.5	21.6 ± 0.3	22.7 ± 0.8
Final body weight (g)	19.1 ± 0.1	16.1 ± 0.7*	18.2 ± 0.6	17.7 ± 0.6	16.1 ± 0.4*
Liver (g/100 g body weight)	4.61 ± 0.13	0.86 ± 0.06*	4.99 ± 0.22	4.95 ± 0.23	5.41 ± 0.21
Lung (g/100 g body weight)	0.79 ± 0.03	0.86 ± 0.07	1.40 ± 0.5	0.81 ± 0.03	0.85 ± 0.07
Kidneys (g/100 g body weight)	1.54 ± 0.06	1.54 ± 0.08	1.47 ± 0.10	1.56 ± 0.07	1.73 ± 0.05
Heart (g/100 g body weight)	0.55 ± 0.02	0.63 ± 0.06	0.53 ± 0.01	0.60 ± 0.03	0.66 ± 0.02

The negative control (CTL) was treated with the vehicle (5% DMSO) used to dilute Ru/5-FU. Data are presented as the mean ± S.E.M. from 5-10 animals. * $P<0.05$ compared to CTL by one-way ANOVA followed by Dunnett's multiple comparisons test.

Table S5. Effect of Ru/5-FU treatment on hematological parameters of peripheral blood of C. B-17 SCID mice inoculated with HCT116 cells

Parameters	CTL	5-FU	DOX	Ru/5-FU	Ru/5-FU
Dose (mg/kg)	-	15	0.8	2	4
Erythrocytes (10^6/mm^3)	9.51 ± 0.19	8.73 ± 0.05	8.87 ± 0.06	9.76 ± 0.15	9.54 ± 0.26
Hemoglobin (g/dL)	12.7 ± 0.5	11.5 ± 0.4	11.3 ± 0.1	13.2 ± 0.3	13.0 ± 0.6
Hematocrit (%)	50.2 ± 2.4	46.6 ± 1,3	44.6 ± 0.7	53.1 ± 1.0	52.3 ± 1.5
MCV (fL)	52.3 ± 1.2	51.3 ± 0.3	51.6 ± 0.4	51.9 ± 0.3	52.4 ± 0.4
Platelets (10^3/mm^3)	187.3 ± 30.1	395.8 ± 19.9*	127.6 ± 38.0	205.8 ± 49.6	301.0 ± 58.0
Leukocytes (10^3/mm^3)	2.04 ± 0.51	0.30 ± 0.04*	2.53 ± 0.17	3.16 ± 0.54	1.47 ± 0.09*
Differential leukocytes (%)					
Lymphocytes	46.1	N.d.	56.1	51.3	35.7
Monocytes	34.5	N.d.	28.8	33.0	43.8
Granulocytes	19.3	N.d.	15.0	15.7	18.8

The negative control (CTL) was treated with the vehicle (5% DMSO) used to dilute the test compound. Data are presented as the mean ± S.E.M. from 5-7 animals. * $P<0.05$ compared to CTL by one-way ANOVA followed by Dunnett's multiple comparisons test. N.d. Not determined.

Table S6. List of cells used

Cells	Histological type	Species	Source[a,b,c]
Cancer cell lines			
HCT116	colorectal carcinoma	human	ATCC
HepG2	hepatocellular carcinoma	human	ATCC
NB4	acute promyelocytic leukemia	human	ATCC
THP-1	monocytic leukemia	human	ATCC
JUKART	T-cell lymphoid leukemia	human	ATCC
K-562	chronic myelogenous leukemia	human	ATCC
HL-60	acute promyelocytic leukemia	human	ATCC
KG-1a	acute myeloid leukemia	human	ATCC
MDA-MB-231	breast carcinoma	human	BCRJ
MCF-7	breast adenocarcinoma	human	ATCC
4T1	breast carcinoma	mouse	ATCC
HSC-3	oral squamous cell carcinoma	human	ATCC
CAL 27	oral squamous cell carcinoma	human	ATCC
SCC-25	oral squamous cell carcinoma	human	ATCC
SCC4	oral squamous cell carcinoma	human	ATCC
SCC-9	oral squamous cell carcinoma	human	ATCC
A549	lung adenocarcinoma	human	BCRJ
NCI-H1299	non-small cell lung carcinoma	human	BCRJ
PANC-1	pancreas ductal adenocarcinoma	human	BCRJ
OVCAR-3	ovarian carcinoma	human	BCRJ
DU 145	prostate carcinoma	human	BCRJ
U-87 MG	glioblastoma	human	BCRJ
A-375	melanoma	human	BCRJ
B16-F10	melanoma	mouse	ATCC
Noncancer cell lines			
MRC-5	lung fibroblast	human	ATCC
BJ	foreskin fibroblast	human	ATCC
Mutant and its parental cell lines			

BAD KO SV40 MEF	immortalized mouse embryonic fibroblasts with the BAD gene knocked out	mouse	ATCC
WT SV40 MEF	wild-type immortalized embryonic fibroblasts	mouse	ATCC
Primary cells			
CR01	colorectal carcinoma	human	primary cell culture
CR02	colorectal carcinoma	human	primary cell culture
CR03	metastatic cholangiocarcinoma	human	primary cell culture
CR04	malignant papillary mesothelioma	human	primary cell culture
PBMC	heath peripheral blood mononuclear cells	human	primary cell culture

[a]ATCC denotes American Type Culture Collection (U.S. and BCRJ denotes the Rio de Janeiro Cell Bank (Brazil). [b]Primary cell cultures of CR01, CR02, CR03 and CR04 were obtained by enzymatic digestion of tumor fragments using trypsin (0.25%) and kept in a water bath at 37°C for 100 minutes. The dissociated fragments were filtered through a 100 µm cell strainer (BD Biosciences, USA). Cells were centrifuged, washed and resuspended in RPMI 1640 or DMEM-F12 medium containing 20% FBS and 1% antibiotic. Cells were plated at 5 x 10^5 cells/well. The Research Ethics Committee of São Rafael Hospital (Salvador, Bahia, Brazil) approved the protocol (CAAE 30815214.9.3002.0048). [c]Primary cell culture of PBMCs was obtained from peripheral blood from healthy donors by a standard Ficoll density protocol. Then, PBMCs were resuspended in RPMI 1640 or DMEM-F12 medium with 20% FBS and 1% antibiotic. Cells were plated at 5 x 10^5 cells/well. Concanavalin A (10 µg/mL, Sigma–Aldrich) was used as a mitogen to trigger cell division in T lymphocytes and was added at the beginning of the culture. The Research Ethics Committee of the Oswaldo Cruz Foundation (Salvador, Bahia, Brazil) approved the protocol (CAAE 16220713.2.0000.0040).

Table S7. List of antibodies used

Epitope	Fluorchrome	Clone	Application	Catalog number	Company
4EBP1 (pT36/pT45)	AF488	M31-16	Intracellular staining	560287	BD Phosflow
Akt (pS473)	AF488	M89-61	Intracellular staining	560404	BD Phosflow
Akt (pT308)	PE	J1-223.371	Intracellular staining	558275	BD Phosflow
Akt 1	PE	55/PKBa/Akt	Intracellular staining	560049	BD Phosflow
CD133	PE	W6B3C1 (W6B3)	Cell surface staining	566594	BD Pharmingen
Cleaved PARP (Asp214)	PE	F21-852	Intracellular staining	552933	BD Pharmingen
E-Cadherin	BV421	36/E-Cadherin	Cell surface staining	564186	BD Horizon
elF4E (pS209)	PE	J77-925	Intracellular staining	560229	BD Phosflow
GSK-3β (pS9)	AF488	D85E12	Intracellular staining	14026S	Cell Signaling Technology
HSP90	PE	C45G5	Intracellular staining	70657S	Cell Signaling Technology
LC3B	AF647	1251A	Intracellular staining	IC9390R	R&D Systems
mTOR (pS2448)	PE	O21-404	Intracellular staining	563489	BD Phosflow
NF-κB p65	PE	14G10A21	Intracellular staining	653004	BioLegend
NF-κB p65 (pS529)	AF488	K10-895.12.50	Intracellular staining	558421	BD Phosflow

NF-κB p65 (pS536)	FITC	NFKBp65 S536-B7	Intracellular staining	MA5-37157	Invitrogen
p62/SQSTM1	AF488	864807	Intracellular staining	IC8028G	R&D Systems
PI3K p85/p55 (pT458/pT199)	PE	PI3KY458-1A11	Intracellular staining	MAS-28027	Invitrogen
S6 (pS235/pS236)	AF488	N7-548	Intracellular staining	560434	BD Phosflow
Vimentin	AF488	RV202	Intracellular staining	562338	BD Pharmingen
IgG1, κ Isotype Control	PE	MOPC-21	Cell surface staining	556650	BD Pharmingen
